# 𝕋-Proper Hypercomplex Centralized Fusion Estimation for Randomly Multiple Sensor Delays Systems with Correlated Noises

**DOI:** 10.3390/s21175729

**Published:** 2021-08-25

**Authors:** Rosa M. Fernández-Alcalá, Jesús Navarro-Moreno, Juan C. Ruiz-Molina

**Affiliations:** Department of Statistics and Operations Research, University of Jaén, Paraje Las Lagunillas, 23071 Jaén, Spain; jnavarro@ujaen.es (J.N.-M.); jcruiz@ujaen.es (J.C.R.-M.)

**Keywords:** centralized fusion estimation, random delay systems, tessarine processing, 𝕋_k_ properness

## Abstract

The centralized fusion estimation problem for discrete-time vectorial tessarine signals in multiple sensor stochastic systems with random one-step delays and correlated noises is analyzed under different T-properness conditions. Based on $T_{k}$, k=1,2, linear processing, new centralized fusion filtering, prediction, and fixed-point smoothing algorithms are devised. These algorithms have the advantage of providing optimal estimators with a significant reduction in computational cost compared to that obtained through a real or a widely linear processing approach. Simulation examples illustrate the effectiveness and applicability of the algorithms proposed, in which the superiority of the $T_{k}$ linear estimators over their counterparts in the quaternion domain is apparent.

## 1. Introduction

Multi-sensor systems and related information fusion estimation theory have attracted much attention over the last few decades due to their wide range of applications in many fields, including target tracking, robotics, navigation, big data, and signal processing [1,2,3,4,5,6,7].

In practice, failures during data transmission are unavoidable and lead to uncertain systems. In this regard, a significant problem is the estimation of the state from systems with random sensor delays (see, for example, ref. [8,9,10,11,12,13]). Such delays may be mainly caused by computational load, heavy network traffic, and the limited bandwidth of the communication channel, as well as other limitations, which mean that the measurements are not always up to date [8]. It is commonly assumed that measurement delays can be described by Bernoulli distributed random variables with known conditional probabilities, where the values 1 and 0 of these variables indicate the presence or absence of measurement delays in the corresponding sensor [10].

Traditionally, there have been two basic approaches to process the information from multiple sensors, centralized and distributed fusion. In the former approach, all the measurement data from each sensor are collected in a fusion center where they are fused and processed, whereas in the distributed fusion method, the measurements of each sensor are transmitted to a local processor where they are independently processed before being transmitted to the fusion center. It is well known that centralized fusion methods lead to the best (optimal) solution when all sensors work healthily [14,15,16]. The strength of this approach lies in the fact that it is easy to implement, and it makes possible the best use of the available information. Accordingly, with the purpose of optimal estimation, centralized fusion methodology has received increased attention in recent literature related to multi-sensor fusion estimation (see, for example, ref. [9,17,18,19]). Notwithstanding the foregoing, the main disadvantage of this approach is the high computational load that may be required, especially when the number of sensors is too large. Alternatively, distributed fusion methodologies are developed with the purpose of designing solutions with a reduced computational load. Although distributed fusion approach presents a better robustness, flexibility and reliability due to its parallel structure; the main handicap of these solutions is that they are suboptimal and, hence, it is desirable to explore other alternatives that can alleviate the computational demand. In this respect, the use of hypercomplex algebras may well offer an ideal framework in which to analyze the properness characteristics of the signals which lead to lower computational costs without losing optimality.

In general, the implementation of hypercomplex algebras in signal processing problems has expanded rapidly because of their natural ability to model multi-dimensional data giving rise to better geometrical interpretations. In this connection, quaternions and tessarines appear as 4D hypercomplex algebras composed of a real part and three imaginary parts, which provide them with the ideal structure for describing three and four-dimensional signals. Nowadays, they play a fundamental role in a variety of applications such as robotics, avionics, 3D graphics, and virtual reality [20]. In principle, the use of quaternions or tessarines means renouncing some of the usual algebraic properties of the real or complex fields. Thus, while quaternion algebra is non-commutative, tessarines become a non-division algebra. These properties make each algebra more appropriate for every specific problem. With this in mind, in [21,22,23,24] the application of these two isodimensional algebras is compared with the objective of showing how the choice of a particular algebra may determine the proposed method performance.

In the related literature, quaternion algebra has been widely used as a signal processing tool and it is still a trending topic in different areas. In particular, in the area of multi-sensor fusion estimation, ref. [25,26] proposed sensor fusion estimation algorithms based on a quaternion extended Kalman filter, ref. [27,28] have provided robust distributed quaternion Kalman filtering algorithm for data fusion over sensor networks dealing with three-dimensional data, and [29] designed a linear quaternion fusion filter from multi-sensor observations. A common characteristic of all the estimation algorithms above is that their methodologies are based on a strictly linear (SL) processing. However, in the quaternion domain, optimal linear processing is widely linear (WL), which requires the consideration of the quaternion signal and its three involutions. In this framework, ref. [30] devised WL filtering, prediction and smoothing algorithms for multi-sensor systems with mixed uncertainties of sensor delays, packet dropout and missing observations. Interestingly, when the signal presents properness properties (cancellation of one or more of the three complementary covariance matrices), the optimal processing is SL (if the signal is Q-proper) or semi-widely linear (if the signal is C-proper), which amounts to operate on a vector with reduced dimension, which means a significant reduction in the computational load of the associated algorithms (please review [31,32,33,34] for further details).

On the other hand, the use of tessarines is less common in the signal processing literature and, to the best of the authors’ knowledge, they have never been considered in multi-sensor fusion estimation problems. In general, the use of tessarines in estimation problems has been limited by the fact that it is not being a normed division algebra. This drawback was successfully overcome in [23] by introducing a metric that guarantees the existence and unicity of the optimal estimator. Moreover, although the optimal processing in the tessarine field is the WL processing, under properness conditions, it is possible to get the optimal solution from estimation algorithms with lower computational costs. In this sense, ref. [23,24] introduced the concept of $T_{1}$ and $T_{2}$-properness and provided a statistical test to determine whether a signal presents one of these properness properties. According to the type of properness, the most suitable form of processing is $T_{1}$ linear processing, which supposes to operate on the signal itself, or $T_{2}$ linear processing, based on the augmented vector given by the signal and its conjugate. The application of both $T_{1}$ and $T_{2}$ linear processing to the estimation problem has provided optimal estimation algorithms of reduced dimension.

Motivated by the above discussions, in this paper we consider a tessarine multiple sensor system where each sensor may be delayed at any time independently from the others. The probability of the occurrence of each delay is dealt by a Bernoulli distribution. Moreover, unlike most sensor fusion estimation algorithms, the observation noises of different sensors can be correlated. In this context, new centralized fusion filtering, prediction and fixed-point smoothing algorithms are designed under both $T_{1}$ and $T_{2}$-properness conditions. The algorithms proposed provide the optimal estimations of the state; meanwhile, the computational load has been reduced with respect to the counterpart tessarine WL (TWL) estimation algorithms. It is important to note that such savings in computational demand cannot be achieved in the real field. The superiority of these algorithms obtained from a $T_{k}$ linear approach over those derived in the quaternion domain is numerically demonstrated under different conditions of properness.

The remainder of the paper is organized as follows. Section 2 introduces the notation used throughout the paper and briefly reviews the main concepts related to the processing of tessarine signals and their implications under $T_{k}$ properness. Then, in Section 3, the problem of estimating a tessarine signal in linear discrete stochastic systems with random state delays and multiple sensors is formulated. Concretely, under $T_{k}$ -properness conditions, a compact state-space model of reduced dimension is proposed. From this model, and based on $T_{k}$-properness properties, $T_{k}$ centralized fusion filtering, step ahead prediction, and fixed-point smoothing algorithms are devised in Section 4. Furthermore, the goodness of these algorithms in performance is numerically analyzed in Section 5 by means of a simulation example, where the superiority of the $T_{k}$ estimation algorithms above over their counterparts in the quaternion domain is evidenced. The paper ends with a section of conclusions. In order to maintain continuity, all technical proofs have been deferred to the Appendixes Appendix A, Appendix B, Appendix C.

## 2. Preliminaries

Throughout this paper, and unless otherwise stated, all the random variables are assumed to have zero-mean. Moreover, the notation and terminology is fairly standard. They are summarized in the following two subsections.

### 2.1. Notation

Matrices are indicated by boldfaced uppercase letters, column vectors as boldfaced lowercase letters, and scalar quantities by lightfaced lowercase letters. Moreover, the following notation is used.

$I_{n}$ denotes the identity matrix of dimension *n*.
$0_{n\times m}$ denotes the n×m zero matrix.
$1_{n}$ denotes the *n*-vector of all ones.
$0_{n}$ denotes the *n*-vector of all zeros.
Superscript * stands for the tessarine conjugate.
Superscript T stands for the transpose.
Superscript H stands for the Hermitian transpose.
Subscript *r* represents the real part of a tessarine.
Subscript ν, for $\nu =\eta ,\eta^{\prime },\eta^{\prime \prime }$, represents the imaginary part of a tessarine.
Z stands for the integer field.
R stands for the real field. Accordingly, $A\in R^{n\times m}$ means that A is a real n×m matrix, and similarly $r\in R^{n}$ means that r is a *n*-dimensional real vector.
T stands for the tessarine field. Accordingly, $A\in T^{n\times m}$ means that A is a tessarine n×m matrix, and similarly $r\in T^{n}$ means that r is a *m*-dimensional real vector.
E[·] is the expectation operator.
Cov(·) is the covariance operator.
diag(·) is a diagonal (or block diagonal) matrix with elements specified on the main diagonal.
$\delta_{n,l}$ is the Kronecker delta function, which is equal to one if l=n, and zero otherwise.
∘ denotes the Hadamard product.
⊗ denotes the Kronecker product.

### 2.2. Basic Concepts and Properties

The following property of the Hadamard product will be useful.

**Property** **1.** *If $A\in R^{n\times n}$ and $b\in R^{n}$, then*

(1) $$\mathrm{diag}\left(b\right)A\mathrm{diag}\left(b\right)=\left(bb^{T}\right)\circ A.$$

**Definition** **1.** *A tessarine random signal $x\left(t\right)\in T^{n}$ is a stochastic process of the form [23]*

$$x\left(t\right)=x_{r}\left(t\right)+\eta x_{\eta }\left(t\right)+\eta^{\prime }x_{\eta^{\prime }}\left(t\right)+\eta^{\prime \prime }x_{\eta^{\prime \prime }}\left(t\right),\;t\in Z$$

*where $x_{\nu }\left(t\right)\in R^{n}$, for $\nu =r,\eta ,\eta^{\prime },\eta^{\prime \prime }$, are real random signals and $\left\{1,\eta ,\eta^{\prime },\eta^{\prime \prime }\right\}$ obeys the following rules:*

$$\begin{array}{c} \eta \eta^{\prime }=\eta^{\prime \prime },\;\eta^{\prime }\eta^{\prime \prime }=\eta ,\;\eta^{\prime \prime }\eta =-\eta^{\prime },\;\eta^{2}={\eta^{\prime \prime }}^{2}=-1,\;{\eta^{\prime }}^{2}=1. \end{array}$$

The conjugate of a given tessarine random signal $x\left(t\right)\in T^{n}$, is

$$x^{*}\left(t\right)=x_{r}\left(t\right)-\eta x_{\eta }\left(t\right)+\eta^{\prime }x_{\eta^{\prime }}\left(t\right)-\eta^{\prime \prime }x_{\eta^{\prime \prime }}\left(t\right).$$

Moreover, the following two auxiliary tessarine vectors are defined:

$$x^{\eta }\left(t\right)=x_{r}\left(t\right)+\eta x_{\eta }\left(t\right)-\eta^{\prime }x_{\eta^{\prime }}\left(t\right)-\eta^{\prime \prime }x_{\eta^{\prime \prime }}\left(t\right),$$

$$x^{\eta^{\prime \prime }}\left(t\right)=x_{r}\left(t\right)-\eta x_{\eta }\left(t\right)-\eta^{\prime }x_{\eta^{\prime }}\left(t\right)+\eta^{\prime \prime }x_{\eta^{\prime \prime }}\left(t\right).$$

For a complete description of the second-order statistical properties of x(t), we need to consider the augmented tessarine signal vector $\bar{x}\left(t\right)={\left[x^{{}^{T}}\left(t\right),x^{H}\left(t\right),x^{\eta^{T}}\left(t\right),x^{\eta^{\prime \prime T}}\left(t\right)\right]}^{T}$. The following relationship between the augmented vector and the real vector $x^{r}\left(t\right)={\left[x_{r}^{T}\left(t\right),x_{\eta }^{T}\left(t\right),x_{\eta^{\prime }}^{T}\left(t\right),x_{\eta^{\prime \prime }}^{T}\left(t\right)\right]}^{T}$ can be established:

$$\bar{x}\left(t\right)=2T_{n}x^{r}\left(t\right),$$

where $T_{n}=\frac{1}{2}A⊗I_{n}$

$$A=\left[\begin{array}{cccc} 1 & \eta & \eta^{\prime } & \eta^{\prime \prime }\\ 1 & -\eta & \eta^{\prime } & -\eta^{\prime \prime }\\ 1 & \eta & -\eta^{\prime } & -\eta^{\prime \prime }\\ 1 & -\eta & -\eta^{\prime } & \eta^{\prime \prime } \end{array}\right],$$

with $T_{n}^{H}T_{n}=I_{4n}$.

**Definition** **2.** *Given two tessarine random signals $x\left(t\right),y\left(s\right)\in T^{n}$, the product ★ between them is defined as*

(2) $$x\left(t\right)★y\left(s\right)=x_{r}\left(t\right)\circ y_{r}\left(s\right)+\eta x_{\eta }\left(t\right)\circ y_{\eta }\left(s\right)+\eta^{\prime }x_{\eta^{\prime }}\left(t\right)\circ y_{\eta^{\prime }}\left(s\right)+\eta^{\prime \prime }x_{\eta^{\prime \prime }}\left(t\right)\circ y_{\eta^{\prime \prime }}\left(s\right).$$

The following property of the product ★ is easy to check.

**Property** **2.** *The augmented vector of x(t)★y(s) is $\bar{x(t)★y(s)}={\bar{D}}^{x}\left(t\right)\bar{y}\left(s\right)$, where ${\bar{D}}^{x}\left(t\right)=T_{n}\mathrm{diag}\left(x^{r}\left(t\right)\right)T_{n}^{H}$.*

**Definition** **3.** *The pseudo autocorrelation function of $x\left(t\right)\in T^{n}$ is defined as $R_{x}\left(t,s\right)=E\left[x\left(t\right)x^{H}\left(s\right)\right]$, ∀t,s∈Z, and the pseudo cross-correlation function of $x\left(t\right),y\left(t\right)\in T^{n}$ is defined as $R_{xy}\left(t,s\right)=E\left[x\left(t\right)y^{H}\left(s\right)\right]$, ∀t,s∈Z.*

Note that, depending on the vanishing of the different *pseudo* correlation functions $R_{xx^{\nu }}\left(t,s\right)$, $\nu =*,\eta ,\eta^{\prime \prime }$, various kinds of tessarine properness can be defined. In particular, the following properness conditions in the tessarine domain have recently been introduced in [23,24].

**Definition** **4.** *A random signal $x\left(t\right)\in T^{n}$ is said to be $T_{1}$-proper (respectively, $T_{2}$-proper) if, and only if, the functions $R_{xx^{\nu }}\left(t,s\right)$, with $\nu =*,\eta ,\eta^{\prime \prime }$ (respectively, $\nu =\eta ,\eta^{\prime \prime }$), vanish ∀t,s∈Z.*

*In a like manner, two random signals $x\left(t\right)\in T^{n_{1}}$ and $y\left(t\right)\in T^{n_{2}}$ are cross $T_{1}$-proper, (respectively, cross $T_{2}$-proper) if, and only if, the functions $R_{xy^{\nu }}\left(t,s\right)$, with $\nu =*,\eta ,\eta^{\prime \prime }$ (respectively, $\nu =\eta ,\eta^{\prime \prime }$), vanish ∀t,s∈Z.*

*Moreover, x(t) and y(t) are jointly $T_{1}$-proper (respectively, jointly $T_{2}$-proper) if, and only if, they are $T_{1}$-proper (respectively, $T_{2}$-proper) and cross $T_{1}$-proper (respectively, cross $T_{2}$-proper).*

Remark that, $T_{1}$ properness is more restrictive than $T_{2}$ properness. Statistical tests to experimentally check whether a signal is $T_{k}$-proper, k=1,2, or improper have been proposed in [23,24].

It should be highlighted that the different properness properties have direct implications on the optimal linear processing. In general, the optimal linear processing is the widely linear processing, which requires to operate on the augmented tessarine vector $\bar{x}\left(t\right)$. Nevertheless, in the case of joint $T_{k}$-properness, k=1,2, the optimal linear processing is reduced to a $T_{k}$ linear processing, with the corresponding decrease in the dimension of the problem. In particular, $T_{1}$ linear processing is based on the tessarine random signal itself, and $T_{2}$ linear processing considers the augmented vector given by the signal and its conjugate [24].

## 3. Problem Formulation

Consider the class of linear discrete stochastic systems with state delays and multiple sensors

(3) $$\begin{array}{cc} x(t+1)= & F_{1}\left(t\right)x\left(t\right)+F_{2}\left(t\right)x^{*}\left(t\right)+F_{3}\left(t\right)x^{\eta }\left(t\right)+F_{4}\left(t\right)x^{\eta^{\prime \prime }}\left(t\right)+u\left(t\right),\;\;t\geq 0\\ z^{\left(i\right)}\left(t\right)= & x\left(t\right)+v^{\left(i\right)}\left(t\right),\;\;t\geq 0,\;\;i=1,\ldots ,R\\ y^{\left(i\right)}\left(t\right)= & \gamma^{\left(i\right)}\left(t\right)★z^{\left(i\right)}\left(t\right)+\left(1_{n}-\gamma^{\left(i\right)}\left(t\right)\right)★z^{\left(i\right)}\left(t-1\right),\;\;t\geq 1,\;\;i=1,\ldots ,R \end{array}$$

where *R* is the number of sensors, ★ is the product defined in (2), $F_{j}\left(t\right)\in T^{n\times n}$, j=1,2,3,4, are deterministic matrices, $x\left(t\right)\in T^{n}$ is the system state to be estimated, $u\left(t\right)\in T^{n}$ is a tessarine noise, $z^{\left(i\right)}\left(t\right)\in T^{n}$ is the *i*th sensor outputs with tessarine sensor noise $v^{\left(i\right)}\left(t\right)\in T^{n}$, $y^{\left(i\right)}\left(t\right)\in T^{n}$ is the observation of the *i*th sensor, $\gamma^{\left(i\right)}\left(t\right)={\left[\gamma_{1}^{\left(i\right)}\left(t\right),\ldots ,\gamma_{n}^{\left(i\right)}\left(t\right)\right]}^{T}\in T^{n}$ is a tessarine random vector with components $\gamma_{j}^{\left(i\right)}\left(t\right)=\gamma_{j,r}^{\left(i\right)}\left(t\right)+\eta \gamma_{j,\eta }^{\left(i\right)}\left(t\right)+\eta^{\prime }\gamma_{j,\eta^{\prime }}^{\left(i\right)}\left(t\right)+\eta^{\prime \prime }\gamma_{j,\eta^{\prime \prime }}^{\left(i\right)}\left(t\right)$, for j=1,…,n, composed of independent Bernoulli random variables $\gamma_{j,\nu }^{\left(i\right)}\left(t\right)$, j=1,⋯,n, $\nu =r,\eta ,\eta^{\prime },\eta^{\prime \prime }$, with known probabilities $p_{j,\nu }^{\left(i\right)}\left(t\right)$, and with possible outcomes {0,1} that indicates if the ν part of the *j*th observation component of the *i*th sensor is up-to-date (case $\gamma_{j,\nu }^{\left(i\right)}\left(t\right))=1$) or there exits one-step delay (case $\gamma_{j,\nu }^{\left(i\right)}\left(t\right))=0$).

The following assumptions for the above system (3) are made.

**Assumption** **1.** *For a given sensor i, the Bernoulli variable vector $\gamma^{\left(i\right)}\left(t\right)$ is independent of $\gamma^{\left(i\right)}\left(s\right)$, for t≠s, and also $\gamma^{\left(i\right)}\left(t\right)$ is independent of $\gamma^{\left(j\right)}\left(t\right)$, for any two sensors i≠j.*

**Assumption** **2.** *For a given sensor i, $\gamma^{\left(i\right)}\left(t\right)$ is independent of x(t), u(t) and $v^{\left(j\right)}\left(t\right)$, for any i,j=1,…,R.*

**Assumption** **3.** *u(t) and $v^{\left(i\right)}\left(t\right)$ are correlated white noises with respective pseudo variances Q(t) and $R^{\left(i\right)}\left(t\right)$. Moreover, $E\left[u\left(t\right)v^{{\left(i\right)}^{H}}\left(s\right)\right]=S^{\left(i\right)}\left(t\right)\delta_{t,s}$.*

**Assumption** **4.** *$v^{\left(i\right)}\left(t\right)$ is independent of $v^{\left(j\right)}\left(t\right)$, for any two sensors i≠j.*

**Assumption** **5.** *The initial state x(0) is independent of the additive noises u(t) and $v^{\left(i\right)}\left(t\right)$, for t≥0 and i=1,…,R.*

**Remark** **1.** *From the hypotheses established on the Bernoulli random variables it follows that, for any $j_{1},j_{2}=1,\ldots ,n$, $\nu_{1},\nu_{2}=r,\eta ,\eta^{\prime },\eta^{\prime \prime }$ and $i_{1},i_{2}=1,\ldots ,R$,*

(4) $$\begin{array}{cc} \; & E\left[\gamma_{j_{1},\nu_{1}}^{\left(i_{1}\right)}\left(t\right)\gamma_{j_{2},\nu_{2}}^{\left(i_{2}\right)}\left(t\right)\right]=\left\{\begin{array}{c} p_{j_{1},\nu_{1}}^{\left(i_{1}\right)}\left(t\right),\;\;if\;i_{1}=i_{2},j_{1}=j_{2},\nu_{1}=\nu_{2}\\ p_{j_{1},\nu_{1}}^{\left(i_{1}\right)}\left(t\right)p_{j_{2},\nu_{2}}^{\left(i_{2}\right)}\left(t\right),\;\;otherwise, \end{array}\right.\\ \; & E\left[\left(1-\gamma_{j_{1},\eta_{1}}^{\left(i_{1}\right)}\left(t\right)\right)\left(1-\gamma_{j_{2},\eta_{2}}^{\left(i_{2}\right)}\left(t\right)\right)\right]=\left\{\begin{array}{c} 1-p_{j_{1},\nu_{1}}^{\left(i_{1}\right)}\left(t\right),\;\;if\;i_{1}=i_{2},j_{1}=j_{2},\nu_{1}=\nu_{2}\\ \left(1-p_{j_{1},\nu_{1}}^{\left(i_{1}\right)}\left(t\right)\right)\left(1-p_{j_{2},\nu_{2}}^{\left(i_{2}\right)}\left(t\right)\right),\;\;otherwise. \end{array}\right. \end{array}$$

### 3.1. One-State Delay System under $T_{k}$-Properness

In this section, a TWL one-state delay system, which exploits the full amount second-order statistics information available, is introduced and analyzed in $T_{k}$-properness scenarios, k=1,2.

For this purpose, consider the augmented vectors $\bar{x}\left(t\right)$, ${\bar{z}}^{\left(i\right)}\left(t\right)$, and ${\bar{y}}^{\left(i\right)}\left(t\right)$ of x(t), $z^{\left(i\right)}\left(t\right)$, and $y^{\left(i\right)}\left(t\right)$, respectively. Then, by applying Property 2 on system (3), the following TWL one-state delay model can be defined:

(5) $$\begin{array}{cc} \bar{x}\left(t+1\right)= & \bar{\Phi }\left(t\right)\bar{x}\left(t\right)+\bar{u}\left(t\right),\;\;t\geq 0\\ {\bar{z}}^{\left(i\right)}\left(t\right)= & \bar{x}\left(t\right)+{\bar{v}}^{\left(i\right)}\left(t\right),\;\;t\geq 0,\;\;i=1,\ldots ,R\\ {\bar{y}}^{\left(i\right)}\left(t\right)= & {\bar{D}}^{\gamma^{\left(i\right)}}\left(t\right){\bar{z}}^{\left(i\right)}\left(t\right)+{\bar{D}}^{\left(1-\gamma^{\left(i\right)}\right)}\left(t\right){\bar{z}}^{\left(i\right)}\left(t-1\right),\;\;t\geq 1,\;\;i=1,\ldots ,R \end{array}$$

where

$$\bar{\Phi }\left(t\right)=\left[\begin{array}{cccc} F_{1}\left(t\right) & F_{2}\left(t\right) & F_{3}\left(t\right) & F_{4}\left(t\right)\\ F_{2}^{*}\left(t\right) & F_{1}^{*}\left(t\right) & F_{4}^{*}\left(t\right) & F_{3}^{*}\left(t\right)\\ F_{3}^{\eta }\left(t\right) & F_{4}^{\eta }\left(t\right) & F_{1}^{\eta }\left(t\right) & F_{2}^{\eta }\left(t\right)\\ F_{4}^{\eta^{\prime \prime }}\left(t\right) & F_{3}^{\eta^{\prime \prime }}\left(t\right) & F_{2}^{\eta^{\prime \prime }}\left(t\right) & F_{1}^{\eta^{\prime \prime }}\left(t\right) \end{array}\right].$$

Moreover, from Assumption 3, the *pseudo* correlation matrices associated to the augmented noise vectors $\bar{u}\left(t\right)$ and ${\bar{v}}^{\left(i\right)}\left(t\right)$ are given by

$E\left[\bar{u}\left(t\right){\bar{u}}^{H}\left(s\right)\right]=\bar{Q}\left(t\right)\delta_{t,s}$;
$E\left[{\bar{v}}^{\left(i\right)}\left(t\right){\bar{v}}^{{\left(i\right)}^{H}}\left(s\right)\right]={\bar{R}}^{\left(i\right)}\left(t\right)\delta_{t,s}$;
$E\left[\bar{u}\left(t\right){\bar{v}}^{{\left(i\right)}^{H}}\left(s\right)\right]={\bar{S}}^{\left(i\right)}\left(t\right)\delta_{t,s}$.

The following result establishes conditions on system (5), which lead to $T_{k}$-properness properties of the processes involved.

**Proposition** **1.** *Consider the TWL one-state delay model (5).*

1. *If x(0) and u(t) are $T_{1}$-proper, and $\bar{\Phi }\left(t\right)$ is a block diagonal matrix of the form*
  $$\bar{\Phi }\left(t\right)=\mathrm{diag}\left(F_{1}\left(t\right),F_{1}^{*}\left(t\right),F_{1}^{\eta }\left(t\right),F_{1}^{\eta^{\prime \prime }}\left(t\right)\right),$$
*then x(t) is $T_{1}$-proper.*
*If additionally $p_{j,r}^{\left(i\right)}\left(t\right)=p_{j,\eta }^{\left(i\right)}\left(t\right)=p_{j,\eta^{\prime }}^{\left(i\right)}\left(t\right)=p_{j,\eta^{\prime \prime }}^{\left(i\right)}\left(t\right)≜p_{j}^{\left(i\right)}\left(t\right)$, ∀t,j,i, $v^{\left(i\right)}\left(t\right)$ is $T_{1}$-proper, and u(t) and $v^{\left(i\right)}\left(t\right)$ are cross $T_{1}$-proper, then x(t) and $y^{\left(i\right)}\left(t\right)$ are jointly $T_{1}$-proper.*
2. *If x(0) and u(t) are $T_{2}$-proper, and $\bar{\Phi }\left(t\right)$ is a block diagonal matrix of the form*
  (6) $$\bar{\Phi }\left(t\right)=\mathrm{diag}\left(\Phi_{2}\left(t\right),\Phi_{2}^{\eta }\left(t\right)\right),\;\;\mathrm{with}\;\;\Phi_{2}\left(t\right)=\left[\begin{array}{cc} F_{1}\left(t\right) & F_{2}\left(t\right)\\ F_{2}^{*}\left(t\right) & F_{1}^{*}\left(t\right) \end{array}\right],$$
*then x(t) is $T_{2}$-proper.*
*If additionally, $p_{j,r}^{\left(i\right)}\left(t\right)=p_{j,\eta }^{\left(i\right)}\left(t\right)$, $p_{j,\eta^{\prime }}^{\left(i\right)}\left(t\right)=p_{j,\eta^{\prime \prime }}^{\left(i\right)}\left(t\right)$, ∀t,j,i, $v^{\left(i\right)}\left(t\right)$ is $T_{2}$-proper and u(t), and $v^{\left(i\right)}\left(t\right)$ are cross $T_{2}$-proper, then x(t) and $y^{\left(i\right)}\left(t\right)$ are jointly $T_{2}$-proper.*

**Proof.**  The proof follows immediately from the application of the corresponding conditions on system (5) and the computation of the augmented *pseudo* correlation matrices $R_{\bar{x}}\left(t,s\right)$ and $R_{\bar{x}{\bar{y}}^{\left(i\right)}}\left(t,s\right)$. □

**Remark** **2.** *Note that under $T_{1}$-properness conditions, ${\bar{\Pi }}^{\gamma^{\left(i\right)}}\left(t\right)=E\left[{\bar{D}}^{\gamma^{\left(i\right)}}\left(t\right)\right]$, i=1,…,R, is a diagonal matrix of the form ${\bar{\Pi }}^{\gamma^{\left(i\right)}}\left(t\right)=I_{4}⊗\Pi_{1}^{\left(i\right)}\left(t\right)$, with $\Pi_{1}^{\left(i\right)}\left(t\right)=\mathrm{diag}\left(p_{1,r}^{\left(i\right)}\left(t\right),\ldots ,p_{n,r}^{\left(i\right)}\left(t\right)\right)$.*

*Likewise, under $T_{2}$-properness conditions, ${\bar{\Pi }}^{\gamma^{\left(i\right)}}\left(t\right)=E\left[{\bar{D}}^{\gamma^{\left(i\right)}}\left(t\right)\right]$, i=1,…,R, takes the form of a block diagonal matrix as follows:*

$${\bar{\Pi }}^{\gamma^{\left(i\right)}}\left(t\right)=\mathrm{diag}\left(\Pi_{2}^{\left(i\right)}\left(t\right),\Pi_{2}^{\left(i\right)}\left(t\right)\right),\;\;\mathrm{with}\;\;\Pi_{2}^{\left(i\right)}\left(t\right)=\frac{1}{2}\left[\begin{array}{cc} \Pi_{a}^{\left(i\right)}\left(t\right) & \Pi_{b}^{\left(i\right)}\left(t\right)\\ \Pi_{b}^{\left(i\right)}\left(t\right) & \Pi_{a}^{\left(i\right)}\left(t\right) \end{array}\right],$$

*where $\Pi_{a}^{\left(i\right)}\left(t\right)=\mathrm{diag}\left(p_{1,r}^{\left(i\right)}\left(t\right)+p_{1,\eta^{\prime }}^{\left(i\right)}\left(t\right),\ldots ,p_{n,r}^{\left(i\right)}\left(t\right)+p_{n,\eta^{\prime }}^{\left(i\right)}\left(t\right)\right)$ and $\Pi_{b}^{\left(i\right)}\left(t\right)=\mathrm{diag}\left(p_{1,r}^{\left(i\right)}\left(t\right)-p_{1,\eta^{\prime }}^{\left(i\right)}\left(t\right),\ldots ,p_{n,r}^{\left(i\right)}\left(t\right)-p_{n,\eta^{\prime }}^{\left(i\right)}\left(t\right)\right)$.*

### 3.2. Compact State-Space Model

By stacking the observations at each sensor in a global observation vector $\vec{z}\left(t\right)={\left[{\bar{z}}^{{\left(1\right)}^{T}}\left(t\right),\ldots ,{\bar{z}}^{{\left(R\right)}^{T}}\left(t\right)\right]}^{T}$, the TWL one-state delay system (5) can be rewritten in a compact form as

(7) $$\begin{array}{cc} \; & \bar{x}\left(t+1\right)=\bar{\Phi }\left(t\right)\bar{x}\left(t\right)+\bar{u}\left(t\right),\;\;t\geq 0\\ \; & \vec{z}\left(t\right)=\bar{C}\bar{x}\left(t\right)+\vec{v}\left(t\right),\;\;t\geq 0\\ \; & \vec{y}\left(t\right)={\bar{D}}^{\vec{\gamma }}\left(t\right)\vec{z}\left(t\right)+{\bar{D}}^{\left(1-\vec{\gamma }\right)}\left(t\right)\vec{z}\left(t-1\right),\;\;t\geq 1 \end{array}$$

where $\vec{v}\left(t\right)$ and $\vec{y}\left(t\right)$ denote the stacking vector of ${\bar{v}}^{{\left(i\right)}^{T}}\left(t\right)$ and ${\bar{y}}^{{\left(i\right)}^{T}}\left(t\right)$, for i=1,…,R, respectively. Moreover, $\bar{C}=1_{R}⊗I_{4n}$, ${\bar{D}}^{\vec{\gamma }}\left(t\right)=\bar{L}\mathrm{diag}\left({\vec{\gamma }}^{r}\left(t\right)\right){\bar{L}}^{H}$ and ${\bar{D}}^{\left(1-\vec{\gamma }\right)}\left(t\right)=\bar{L}\mathrm{diag}\left(1_{4Rn}-{\vec{\gamma }}^{r}\left(t\right)\right){\bar{L}}^{H}$, with $\bar{L}=I_{R}⊗T_{n}$.

In addition, $E\left[\vec{v}\left(t\right){\vec{v}}^{H}\left(s\right)\right]=\bar{R}\left(t\right)\delta_{t,s}$, with $\bar{R}\left(t\right)=\mathrm{diag}\left({\bar{R}}^{\left(1\right)}\left(t\right),\ldots ,{\bar{R}}^{\left(R\right)}\left(t\right)\right)$, and $E\left[\bar{u}\left(t\right){\vec{v}}^{H}\left(s\right)\right]=\bar{S}\left(t\right)\delta_{t,s}$, with $\bar{S}\left(t\right)=\left[{\bar{S}}^{\left(1\right)}\left(t\right),\ldots ,{\bar{S}}^{\left(R\right)}\left(t\right)\right]$.

In this paper, our aim is to investigate the centralized fusion estimation problem under conditions of $T_{k}$-properness, with k=1,2. In this sense, the use of $T_{k}$-properness properties allows us to consider the observation equation with reduced dimension

(8) $$\begin{array}{c} y_{k}\left(t\right)={\tilde{D}}_{k}^{\vec{\gamma }}\left(t\right)\bar{C}\bar{x}\left(t\right)+{\tilde{D}}_{k}^{\left(1-\vec{\gamma }\right)}\left(t\right)\bar{C}\bar{x}\left(t-1\right)+{\tilde{D}}_{k}^{\vec{\gamma }}\left(t\right)\vec{v}\left(t\right)+{\tilde{D}}_{k}^{\left(1-\vec{\gamma }\right)}\left(t\right)\vec{v}\left(t-1\right),\;t\geq 1 \end{array}$$

where $\bar{x}\left(t\right)$ satisfies the state equation in (7), ${\tilde{D}}_{k}^{\vec{\gamma }}\left(t\right)=L_{k}\mathrm{diag}\left({\vec{\gamma }}^{r}\left(t\right)\right){\bar{L}}^{H}$ and ${\tilde{D}}_{k}^{\left(1-\vec{\gamma }\right)}\left(t\right)=L_{k}\mathrm{diag}\left(1_{4Rn}-{\vec{\gamma }}^{r}\left(t\right)\right){\bar{L}}^{H}$, with $L_{k}=I_{R}⊗T_{k}$ and $T_{k}=\frac{1}{2}B_{k}⊗I_{n}$, where

- $T_{1}$-proper scenario:
  $B_{1}=\left[\begin{array}{cccc} 1 & \eta & \eta^{\prime } & \eta^{\prime \prime } \end{array}\right]$;
  $y_{1}\left(t\right)≜{\left[y^{{\left(1\right)}^{T}}\left(t\right),\ldots ,y^{{\left(R\right)}^{T}}\left(t\right)\right]}^{T}$.
- $T_{2}$-proper scenario:
  $B_{2}=\left[\begin{array}{cccc} 1 & \eta & \eta^{\prime } & \eta^{\prime \prime }\\ 1 & -\eta & \eta^{\prime } & -\eta^{\prime \prime } \end{array}\right]$;
  $y_{2}\left(t\right)≜{\left[y^{{\left(1\right)}^{T}}\left(t\right),y^{{\left(1\right)}^{H}}\left(t\right),\ldots ,y^{{\left(R\right)}^{T}}\left(t\right),y^{{\left(R\right)}^{H}}\left(t\right)\right]}^{T}$.

**Remark** **3.** *Note that under $T_{k}$-properness conditions, ${\tilde{\Pi }}_{k}^{\vec{\gamma }}\left(t\right)=E\left[{\tilde{D}}_{k}^{\vec{\gamma }}\left(t\right)\right]$ is given by ${\tilde{\Pi }}_{k}^{\vec{\gamma }}\left(t\right)=\mathrm{diag}\left({\tilde{\Pi }}_{k}^{\gamma^{\left(1\right)}}\left(t\right),\ldots ,{\tilde{\Pi }}_{k}^{\gamma^{\left(R\right)}}\left(t\right)\right)$, where ${\tilde{\Pi }}_{k}^{\gamma^{\left(i\right)}}\left(t\right)=\left[\Pi_{k}^{\left(i\right)}\left(t\right),0_{kn\times (4-k)n}\right]$ with $\Pi_{k}^{\left(i\right)}\left(t\right)$, i=1,⋯,R, given in Remark 2.*

*Similarly, ${\tilde{\Pi }}_{k}^{\left(1-\vec{\gamma }\right)}\left(t\right)=E\left[{\tilde{D}}_{k}^{\left(1-\vec{\gamma }\right)}\left(t\right)\right]$ is given by the block diagonal matrix ${\tilde{\Pi }}_{k}^{\left(1-\vec{\gamma }\right)}\left(t\right)=\mathrm{diag}\left({\tilde{\Pi }}_{k}^{\left(1-\gamma^{\left(1\right)}\right)}\left(t\right),\ldots ,{\tilde{\Pi }}_{k}^{\left(1-\gamma^{\left(R\right)}\right)}\left(t\right)\right)$ with ${\tilde{\Pi }}_{k}^{\left(1-\gamma^{\left(i\right)}\right)}\left(t\right)=\left[I_{kn}-\Pi_{k}^{\left(i\right)}\left(t\right),0_{kn\times (4-k)n}\right]$.*

Accordingly, whereas the optimal linear processing for the estimation of a tessarine signal x(t) is the TWL processing based on the set of measurements $\left\{\vec{y}\left(1\right),\ldots \vec{y}\left(t\right)\right\}$, under $T_{k}$-properness conditions the optimal estimator of $x\left(t\right)\in T^{n}$, ${\hat{x}}^{T_{k}}\left(t|s\right)$, can be computed by projecting on the set of measurements $\left\{y_{k}\left(1\right),\ldots ,y_{k}\left(s\right)\right\}$, for k=1,2. Thereby, $T_{k}$ estimators are obtained that have the same performance as TWL estimators, but with a lower computational complexity. More importantly, this computational load saving cannot be achieved with the real approach.

Note that tessarine algebra is not a Hilbert space and, as a consequence, neither the existence nor the uniqueness of the projection on a set of tessarines is guaranteed. Nevertheless, this drawback has been overcome in [23] by defining a suitable metric, which assures the existence and uniqueness of these projections.

The following property sets the correlations between the noises, $\bar{u}\left(t\right)$ and $\vec{v}\left(t\right)$, and both the augmented state $\bar{x}\left(t\right)$ and the observations $y_{k}\left(t\right)$.

**Property** **3.** *Under Assumptions 1–4, the following correlations hold.*

1. *Correlations between noises and the augmented state:*
   *(a)*  *$E\left[\bar{x}\left(t+1\right){\bar{u}}^{H}\left(t\right)\right]=\bar{Q}\left(t\right)$;*
   *(b)*  *$E\left[\bar{x}\left(t\right){\bar{u}}^{H}\left(s\right)\right]=0_{4n\times 4n}$, for t≤s;*
   *(c)*  *$E\left[\bar{x}\left(t+1\right){\vec{v}}^{H}\left(t\right)\right]=\bar{S}\left(t\right)$;*
   *(d)*  *$E\left[{\bar{x\left(t\right)\vec{v}}}^{H}\left(s\right)\right]=0_{4n\times 4Rn}$, for t≤s.*
2. *Correlations between noises and $T_{k}$ observations:*
   *(a)*  *$E\left[y_{k}\left(t\right){\bar{u}}^{H}\left(t\right)\right]={\tilde{\Pi }}_{k}^{\vec{\gamma }}\left(t\right){\bar{S}}^{H}\left(t\right)$;*
   *(b)*  *$E\left[y_{k}\left(t+1\right){\bar{u}}^{H}\left(t\right)\right]={\tilde{\Pi }}_{k}^{\vec{\gamma }}\left(t+1\right)\bar{C}\bar{Q}\left(t\right)+{\tilde{\Pi }}_{k}^{\left(1-\gamma \right)}\left(t+1\right){\bar{S}}^{H}\left(t\right)$;*
   *(c)*  *$E\left[y_{k}\left(t\right){\bar{u}}^{H}\left(s\right)\right]=0_{kRn\times 4n}$, for t<s;*
   *(d)*  *$E\left[y_{k}\left(t\right){\vec{v}}^{H}\left(t\right)\right]={\tilde{\Pi }}_{k}^{\vec{\gamma }}\left(t\right)\bar{R}\left(t\right)$;*
   *(e)*  *$E\left[y_{k}\left(t+1\right){\vec{v}}^{H}\left(t\right)\right]={\tilde{\Pi }}_{k}^{\vec{\gamma }}\left(t+1\right)\bar{C}\bar{S}\left(t\right)+{\tilde{\Pi }}_{k}^{\left(1-\gamma \right)}\left(t+1\right)\bar{R}\left(t\right)$;*
   *(f)*  *$E\left[y_{k}\left(t\right){\vec{v}}^{H}\left(s\right)\right]=0_{kRn\times 4Rn}$, for t<s.*

**Remark** **4.** *Observe that, under a $T_{k}$-properness setting, the state equation in (7) is equivalent to the $T_{k}$ state equation*

(9) $$x_{k}\left(t+1\right)=\Phi_{k}\left(t\right)x_{k}\left(t\right)+u_{k}\left(t\right),\;\;t\geq 0$$

*where,*

- *in a $T_{1}$-proper scenario, $x_{1}\left(t\right)≜x\left(t\right)$, $u_{1}\left(t\right)≜u\left(t\right)$, and $\Phi_{1}\left(t\right)≜F_{1}\left(t\right)$;*
- *in a $T_{2}$-proper scenario, $x_{2}\left(t\right)≜{\left[x^{T}\left(t\right),x^{H}\left(t\right)\right]}^{T}$, $u_{2}\left(t\right)≜{\left[u^{T}\left(t\right),u^{H}\left(t\right)\right]}^{T}$ and $\Phi_{2}\left(t\right)$ is as in (6).*

*In such cases, $Q_{k}\left(t\right)=E\left[u_{k}\left(t\right)u_{k}^{H}\left(t\right)\right]$ and $S_{k}\left(t\right)=E\left[u_{k}\left(t\right)v_{k}^{H}\left(t\right)\right]$, for k=1,2, where $v_{1}\left(t\right)≜\vec{v}\left(t\right)$ and $v_{2}\left(t\right)≜{\left[{\vec{v}}^{T}\left(t\right),{\vec{v}}^{H}\left(t\right)\right]}^{T}$, with $\vec{v}\left(t\right)={\left[v^{{\left(1\right)}^{T}}\left(t\right),\ldots ,v^{{\left(R\right)}^{T}}\left(t\right)\right]}^{T}$.*

*Nevertheless, Equation (9) cannot be used together with the observation Equation (8), since the latter involves the augmented state vector $\bar{x}\left(t\right)$.*

## 4. $T_{k}$-Proper Centralized Fusion Estimation Algorithms

In this section, the $T_{k}$ centralized fusion filter, prediction, and fixed-point smoothing algorithms are designed on the basis of the set of observations $\left\{y_{k}\left(1\right),\ldots ,y_{k}\left(s\right)\right\}$, k=1,2, defined in (8).

With this purpose in mind, the observation Equation (8) is used to devise filtering, prediction, and smoothing algorithms for the augmented state vector $\bar{x}\left(t\right)$. Then, by applying $T_{k}$-properness properties, the recursive formulas for the filtering, prediction, and smoothing estimators of $x_{k}\left(t\right)$ are easily determined. Finally, the desired $T_{k}$ centralized fusion filtering, prediction and fixed-point smoothing estimators are obtained as a subvector of them.

Theorems 1–3 summarize the recursive formulas for the computation of these $T_{k}$ estimators as well as their associated error variances.

### 4.1. $T_{k}$ Centralized Fusion Filter

**Theorem** **1.** *The optimal $T_{k}$ centralized fusion filter ${\hat{x}}^{T_{k}}\left(t|t\right)$ and one-step predictor ${\hat{x}}^{T_{k}}\left(t+1|t\right)$ for the state x(t) are obtained by extracting the first n components of the optimal estimator ${\hat{x}}_{k}\left(t|t\right)$ and ${\hat{x}}_{k}\left(t+1|t\right)$, respectively, which are recursively computed from the expressions*

(10) $$\begin{array}{ccc} & & {\hat{x}}_{k}\left(t|t\right)={\hat{x}}_{k}\left(t|t-1\right)+L_{k}\left(t\right)\varepsilon_{k}\left(t\right),\;t\geq 1 \end{array}$$

(11) $$\begin{array}{ccc} & & {\hat{x}}_{k}\left(t+1|t\right)=\Phi_{k}\left(t\right){\hat{x}}_{k}\left(t|t\right)+H_{k}\left(t\right)\varepsilon_{k}\left(t\right),\;t\geq 1 \end{array}$$

*with ${\hat{x}}_{k}\left(0|0\right)=0_{kn}$ and ${\hat{x}}_{k}\left(1|0\right)=0_{kn}$, and where $H_{k}\left(t\right)=S_{k}\left(t\right)\Pi_{k}\left(t\right)\Omega_{k}^{-1}\left(t\right)$, with $\Pi_{k}\left(t\right)=\mathrm{diag}\left(\Pi_{k}^{\left(1\right)}\left(t\right),\ldots ,\Pi_{k}^{\left(R\right)}\left(t\right)\right)$ and $\Pi_{k}^{\left(i\right)}\left(t\right)$, i=1,⋯,R, defined in Remark 2 for k=1,2.* *Moreover, $\varepsilon_{k}\left(t\right)$ are the innovations calculated as follows*

(12) $$\begin{array}{c} \varepsilon_{k}\left(t\right)=y_{k}\left(t\right)-\Pi_{k}\left(t\right)C_{k}{\hat{x}}_{k}\left(t|t-1\right)-\left(I_{m}-\Pi_{k}\left(t\right)\right)C_{k}{\hat{x}}_{k}\left(t-1|t-1\right)\\ -\left(I_{m}-\Pi_{k}\left(t\right)\right)G_{k}\left(t-1\right)\varepsilon_{k}\left(t-1\right),\;t\geq 1 \end{array}$$

*with m=kRn, $\varepsilon_{k}\left(0\right)=0_{m}$, and where $C_{k}=1_{R}⊗I_{kn}$, $G_{k}\left(t\right)=R_{k}\left(t\right)\Pi_{k}\left(t\right)\Omega_{k}^{-1}\left(t\right)$, with $R_{k}\left(t\right)=E\left[v_{k}\left(t\right)v_{k}^{H}\left(t\right)\right]$.*

*In addition, $L_{k}\left(t\right)=\Theta_{k}\left(t\right)\Omega_{k}^{-1}\left(t\right)$, where Θ(t) is computed through the equation*,

(13) $$\begin{array}{c} \Theta_{k}\left(t\right)=P_{k}\left(t|t-1\right)C_{k}^{T}\Pi_{k}\left(t\right)+\Phi_{k}\left(t-1\right)P_{k}\left(t-1|t-1\right)C_{k}^{T}\left(I_{m}-\Pi_{k}\left(t\right)\right)\\ +S_{k}\left(t-1\right)\left(I_{m}-\Pi_{k}\left(t\right)\right)-H_{k}\left(t-1\right)\Theta_{k}^{H}\left(t-1\right)C_{k}^{T}\left(I_{m}-\Pi_{k}\left(t\right)\right)\\ -\Phi_{k}\left(t-1\right)\Theta_{k}\left(t-1\right)G_{k}^{H}\left(t-1\right)\left(I_{m}-\Pi_{k}\left(t\right)\right)\\ -H_{k}\left(t-1\right)\Omega_{k}\left(t-1\right)G_{k}^{H}\left(t-1\right)\left(I_{m}-\Pi_{k}\left(t\right)\right),\;t>1 \end{array}$$

*with $\Theta_{k}\left(1\right)=P_{k}\left(1|0\right)C_{k}^{T}\Pi_{k}\left(1\right)+\Phi_{k}\left(0\right)P_{k}\left(0|0\right)C_{k}^{T}\left(I_{m}-\Pi_{k}\left(1\right)\right)+S_{k}\left(0\right)\left(I_{m}-\Pi_{k}\left(1\right)\right)$, and the innovations covariance matrix $\Omega_{k}\left(t\right)$ is obtained as*

(14) $$\begin{array}{c} \Omega_{k}\left(t\right)=M_{k}^{1}\left(t\right)-M_{k}^{2}\left(t\right)-M_{k}^{3}\left(t\right)+M_{k}^{4}\left(t\right)+\Pi_{k}\left(t\right)C_{k}P_{k}\left(t|t-1\right)C_{k}^{T}\Pi_{k}\left(t\right)\\ +\Pi_{k}\left(t\right)J_{k}\left(t-1\right)\left(I_{m}-\Pi_{k}\left(t\right)\right)+\left(I_{m}-\Pi_{k}\left(t\right)\right)J_{k}^{H}\left(t-1\right)\Pi_{k}\left(t\right)\\ +\left(I_{m}-\Pi_{k}\left(t\right)\right)[C_{k}P_{k}\left(t-1|t-1\right)C_{k}^{T}-C_{k}\Theta_{k}\left(t-1\right)G_{k}^{H}\left(t-1\right)-G_{k}\left(t-1\right)\Theta_{k}^{H}\left(t-1\right)C_{k}^{T}\\ -G_{k}\left(t-1\right)\Omega_{k}\left(t-1\right)\;G_{k}^{H}\left(t-1\right)]\left(I_{m}-\Pi_{k}\left(t\right)\right),\;t>1 \end{array}$$

*with*

(15) $$\begin{array}{c} \Omega_{k}\left(1\right)=M_{k}^{1}\left(1\right)-M_{k}^{2}\left(1\right)-M_{k}^{3}\left(1\right)+M_{k}^{4}\left(1\right)+\Pi_{k}\left(1\right)C_{k}P_{k}\left(1|0\right)C_{k}^{T}\Pi_{k}\left(1\right)\\ +\Pi_{k}\left(1\right)J_{k}\left(0\right)\left(I_{m}-\Pi_{k}\left(1\right)\right)+\left(I_{m}-\Pi_{k}\left(1\right)\right)J_{k}^{H}\left(0\right)\Pi_{k}\left(1\right)\\ +\left(I_{m}-\Pi_{k}\left(1\right)\right)C_{k}P_{k}\left(0|0\right)C_{k}^{T}\left(I_{m}-\Pi_{k}\left(1\right)\right), \end{array}$$

*where*

$$\begin{array}{c} J_{k}\left(t\right)=C_{k}[\Phi_{k}\left(t\right)P_{k}\left(t|t\right)C_{k}^{T}-H_{k}\left(t\right)\Theta_{k}^{H}\left(t\right)C_{k}^{T}+S_{k}\left(t\right)-\Phi_{k}\left(t\right)\Theta_{k}\left(t\right)G_{k}^{H}\left(t\right)\\ -H_{k}\left(t\right)\Omega_{k}\left(t\right)G_{k}^{H}\left(t\right)], \end{array}$$

*with $J_{k}\left(0\right)=C_{k}\left[\Phi_{k}\left(0\right)P_{k}\left(0|0\right)C_{k}^{T}+S_{k}\left(0\right)\right]$, and*

*$M_{k}^{1}\left(t\right)=L_{k}\left\{\mathrm{Cov}\left({\vec{\gamma }}^{r}\left(t\right)\right)\circ \left({\bar{L}}^{H}\bar{C}\bar{\Sigma }\left(t-1\right){\bar{C}}^{T}\bar{L}\right)\right\}L_{k}^{H}$,*
*$M_{k}^{2}\left(t\right)=L_{k}\left\{\mathrm{Cov}\left({\vec{\gamma }}^{r}\left(t\right)\right)\circ \left({\bar{L}}^{H}\bar{C}\bar{S}\left(t\right)\bar{L}\right)\right\}L_{k}^{H}$,*
*$M_{k}^{3}\left(t\right)=L_{k}\left\{\mathrm{Cov}\left({\vec{\gamma }}^{r}\left(t\right)\right)\circ \left({\bar{L}}^{H}{\bar{S}}^{H}\left(t\right){\bar{C}}^{T}\bar{L}\right)\right\}L_{k}^{H}$,*
*$M_{k}^{4}\left(t\right)=L_{k}\left\{\Delta^{p^{r}}\left(t\right)\circ \left({\bar{L}}^{H}\bar{R}\left(t\right)\bar{L}\right)+\Delta^{1-p^{r}}\left(t\right)\circ \left({\bar{L}}^{H}\bar{R}\left(t-1\right)\bar{L}\right)\right\}L_{k}^{H}$,*

*where $\Delta^{p^{r}}\left(t\right)=E\left[{\vec{\gamma }}^{r}\left(t\right){\vec{\gamma }}^{r^{T}}\left(t\right)\right]$, $\Delta^{1-p^{r}}\left(t\right)=E\left[\left(1_{4Rn}-{\vec{\gamma }}^{r}\left(t\right)\right){\left(1_{4Rn}-{\vec{\gamma }}^{r}\left(t\right)\right)}^{T}\right]$, with entries given in (4), and*

$$\bar{\Sigma }\left(t\right)=\left[\bar{\Phi }\left(t\right)\bar{D}\left(t\right){\bar{\Phi }}^{H}\left(t\right)+\bar{Q}\left(t\right)-\bar{\Phi }\left(t\right)\bar{D}\left(t\right)-\bar{D}\left(t\right){\bar{\Phi }}^{H}\left(t\right)+\bar{D}\left(t\right)\right],$$

*where $\bar{D}\left(t\right)=R_{\bar{x}}\left(t,t\right)$ is recursively computed from*

(16) $$\bar{D}\left(t\right)=\bar{\Phi }\left(t-1\right)\bar{D}\left(t-1\right){\bar{\Phi }}^{H}\left(t-1\right)+\bar{Q}\left(t-1\right).$$

*Finally, the*$T_{k}$*filtering and prediction error pseudo covariance matrices*$P^{T_{k}}\left(t|t\right)$*and*$P^{T_{k}}\left(t+1|t\right)$, *respectively, are obtained from the filtering and prediction error pseudo covariance matrices*$P_{k}\left(t|t\right)$*and*$P_{k}\left(t+1|t\right)$*, calculated from the recursive expressions*

(17) $$P_{k}\left(t|t\right)=P_{k}\left(t|t-1\right)-\Theta_{k}\left(t\right)\Omega_{k}^{-1}\left(t\right)\Theta_{k}^{H}\left(t\right),$$

*with $P_{k}\left(0|0\right)=E\left[x_{k}\left(0\right)x_{k}^{H}\left(0\right)\right]$, and*

(18) $$\begin{array}{c} P_{k}\left(t+1|t\right)=\Phi_{k}\left(t\right)P_{k}\left(t|t\right)\Phi_{k}^{H}\left(t\right)-H_{k}\left(t\right)\Theta_{k}^{H}\left(t\right)\Phi_{k}^{H}\left(t\right)\\ -\Phi_{k}\left(t\right)\Theta_{k}\left(t\right)H_{k}^{H}\left(t\right)-H_{k}\left(t\right)\Omega_{k}\left(t\right)H_{k}^{H}\left(t\right)+Q_{k}\left(t\right), \end{array}$$

*with $P_{k}\left(1|0\right)=\Phi_{k}\left(0\right)P_{k}\left(0|0\right)\Phi_{k}^{H}\left(0\right)+Q_{k}\left(0\right)$.*

**Remark** **5.** *In the implementation of the above algorithm, the particular structure of* $\bar{\Sigma }\left(t\right)$ *under* $T_{k}$ *-properness conditions should be taken into consideration. In this regard, it is not difficult to check that* $\bar{\Sigma }\left(t\right)$ *is a block diagonal matrix of the form*

- *$T_{1}$-properness: $\bar{\Sigma }\left(t\right)=\mathrm{diag}\left(\Sigma_{1}\left(t\right),\Sigma_{1}^{*}\left(t\right),\Sigma_{1}^{\eta }\left(t\right),\Sigma_{1}^{\eta^{\prime \prime }}\left(t\right)\right)$;*
- *$T_{2}$-properness: $\bar{\Sigma }\left(t\right)=\mathrm{diag}\left(\Sigma_{2}\left(t\right),\Sigma_{2}^{\eta }\left(t\right)\right)$,*

*with $\Sigma_{k}\left(t\right)=\Phi_{k}\left(t\right)D_{k}\left(t\right)\Phi_{k}^{H}\left(t\right)+Q_{k}\left(t\right)-\Phi_{k}\left(t\right)D_{k}\left(t\right)-D_{k}\left(t\right)\Phi_{k}^{H}\left(t\right)+D_{k}\left(t\right)$, k=1,2, where $D_{k}\left(t\right)=R_{x_{k}}\left(t,t\right)$ is recursively computed from*

$$D_{k}\left(t\right)=\Phi_{k}\left(t-1\right)D_{k}\left(t-1\right)\Phi_{k}^{H}\left(t-1\right)+Q_{k}\left(t-1\right).$$

### 4.2. $T_{k}$ Centralized Fusion Predictor

**Theorem** **2.** *The optimal $T_{k}$ centralized fusion predictor ${\hat{x}}^{T_{k}}\left(t+\tau |t\right)$ for the state x(t) is obtained by extracting the first n components of the optimal estimator ${\hat{x}}_{k}\left(t+\tau |t\right)$, which is recursively computed from the expression*

(19) $${\hat{x}}_{k}\left(t+\tau |t\right)=\Phi_{k}\left(t+\tau -1\right){\hat{x}}_{k}\left(t+\tau -1|t\right),\;\tau \geq 2$$

*with the initialization the one-step predictor ${\hat{x}}_{k}\left(t+1|t\right)$ given by (11).*

*Moreover, the $T_{k}$-proper prediction error pseudo covariance matrix $P^{T_{k}}\left(t+\tau |t\right)$ is obtained from the prediction error pseudo covariance matrix $P_{k}\left(t+\tau |t\right)$, computed from the recursive expression*

(20) $$\begin{array}{c} P_{k}\left(t+\tau |t\right)=\Phi_{k}\left(t+\tau -1\right)P_{k}\left(t+\tau -1|t\right)\Phi_{k}^{H}\left(t+\tau -1\right)+Q_{k}\left(t+\tau -1\right),\;\tau \geq 2 \end{array}$$

*with the initialization the one-step prediction error pseudo covariance matrix given by (18).*

### 4.3. $T_{k}$ Centralized Fusion Smoother

**Theorem** **3.** *The optimal $T_{k}$ centralized fusion fixed-point smoother ${\hat{x}}^{T_{k}}\left(t|s\right)$, for a fixed instant t<s, for the state x(t) is obtained by extracting the n first components of the optimal estimator ${\hat{x}}_{k}\left(t|s\right)$, which is recursively computed from the expressions*

(21) $$\begin{array}{ccc} & & {\hat{x}}_{k}\left(t|s\right)={\hat{x}}_{k}\left(t|s-1\right)+L_{k}\left(t,s\right)\varepsilon_{k}\left(s\right),\;t\geq 1 \end{array}$$

*with initial condition ${\hat{x}}_{k}\left(t|t\right)$ given by (10), and where the innovations $\varepsilon_{k}\left(s\right)$ are recursively computed from (12) and $L_{k}\left(t,s\right)=\Theta_{k}\left(t,s\right)\Omega_{k}^{-1}\left(s\right)$ with $\Omega_{k}^{-1}\left(s\right)$ obtained from the recursive expression (14) and*

(22) $$\begin{array}{c} \Theta_{k}\left(t,s\right)=\left[E_{k}\left(t,s-1\right)\Phi_{k}^{H}\left(s-1\right)-\Theta_{k}\left(t,s-1\right)H_{k}^{H}\left(s-1\right)\right]C_{k}^{T}\Pi_{k}\left(s\right)\\ +\left[E_{k}\left(t,s-1\right)C_{k}^{T}-\Theta_{k}\left(t,s-1\right)G_{k}^{H}\left(s-1\right)\right]\left(I_{m}-\Pi_{k}\left(s\right)\right), \end{array}$$

(23) $$\begin{array}{c} E_{k}\left(t,s\right)=\left[E_{k}\left(t,s-1\right)\Phi_{k}^{H}\left(s-1\right)-\Theta_{k}\left(t,s-1\right)H_{k}^{H}\left(s-1\right)\right]\left(I-C_{k}^{T}\Pi_{k}\left(s\right)L_{k}^{H}\left(s\right)\right)\\ -\left[E_{k}\left(t,s-1\right)C_{k}^{T}-\Theta_{k}\left(t,s-1\right)G_{k}^{H}\left(s-1\right)\right]\left(I_{m}-\Pi_{k}\left(s\right)\right)L_{k}^{H}\left(s\right), \end{array}$$

*with initialization $\Theta_{k}\left(t,t\right)=\Theta_{k}\left(t\right)$ given by (13) and $E_{k}\left(t,t\right)=P_{k}\left(t|t\right)$.*

*Furthermore, the $T_{k}$ fixed-point smoothing error pseudo covariance matrix is recursively computed through the expression*

(24) $$P_{k}\left(t|s\right)=P_{k}\left(t|s-1\right)-\Theta_{k}\left(t,s\right)\Omega_{k}^{-1}\left(s\right)\Theta_{k}^{H}\left(t,s\right),$$

*with $P_{k}\left(t|t\right)$ the filtering error pseudo covariance matrix (17).*

As mentioned above, the main advantage of the proposed $T_{k}$ centralized fusion algorithms is that the resulting $T_{k}$ centralized fusion estimators coincide with the optimal TWL counterparts; meanwhile, they lead to computational savings with respect to the one derived from a TWL approach.

**Remark** **6.** *The computational demand of the proposed tessarine estimation algorithms under $T_{k}$, for k=1,2 properness conditions is similar to that of their counterparts in the quaternion domain, i.e., the QSL and QSWL estimation algorithms, respectively, (review [34] for a comparative analysis of the computational complexity of quaternion estimators). Therefore, the computational load of TWL estimation algorithms is of order $O(64R^{3}n^{3})$, whereas the $T_{k}$, for k=1,2, algorithms are of order $O(m^{3})$, with m=kRn.*

## 5. Simulation Examples

In this section, the effectiveness of the above $T_{k}$-proper centralized fusion estimation algorithms is experimentally analyzed. With this aim, the following simulation examples have be chosen to reveal the superiority of the proposed $T_{k}$-proper estimators over their counterparts in the quaternion domain, when $T_{k}$-properness conditions are present.

Let us consider the following tessarine system with three sensors:

$$\begin{array}{cc} x(t+1)= & f_{1}x\left(t\right)+u\left(t\right)\\ z^{\left(i\right)}\left(t\right)= & x\left(t\right)+v^{\left(i\right)}\left(t\right),\;i=1,2,3\\ y^{\left(i\right)}\left(t\right)= & \gamma^{\left(i\right)}\left(t\right)★z^{\left(i\right)}\left(t\right)+\left(1-\gamma^{\left(i\right)}\left(t\right)\right)★z^{\left(i\right)}\left(t-1\right),\;i=1,2,3 \end{array}$$

with $f_{1}=0.9-0.3\eta +0.02\eta^{\prime }+0.1\eta^{\prime \prime }\in T$. The following assumptions are made on the initial state and additive noises.

1. The initial state $x_{0}$ is a tessarine Gaussian variable determined by the real covariance matrix
  (25) $$E\left[x^{r}\left(0\right)x^{r^{T}}\left(0\right)\right]=\left[\begin{array}{cccc} a & 0 & -2.5 & 0\\ 0 & 4 & 0 & -2.5\\ -2.5 & 0 & a & 0\\ 0 & -2.5 & 0 & 4 \end{array}\right].$$
2. u(t) is a tessarine white Gaussian noise with a real covariance matrix
  (26) $$E\left[u^{r}\left(t\right)u^{r^{T}}\left(s\right)\right]=\left[\begin{array}{cccc} 0.9 & 0 & c & 0\\ 0 & b & 0 & c\\ c & 0 & 0.9 & 0\\ 0 & c & 0 & b \end{array}\right]\delta_{t,s}.$$
3. The measurement noises $v^{\left(i\right)}\left(t\right)$ of the three sensors are tessarine white Gaussian noises defined as
  $$v^{\left(i\right)}\left(t\right)=\alpha_{i}u\left(t\right)+w^{\left(i\right)}\left(t\right),$$
  where the coefficients $\alpha_{i}$ are the constant scalars $\alpha_{1}=0.5$, $\alpha_{2}=0.8$, and $\alpha_{3}=0.4$ and $w^{\left(i\right)}\left(t\right)$, i=1,2,3, are $T_{1}$-proper tessarine white Gaussian noises with mean zeros and real covariance matrices
  $$E\left[w^{{\left(i\right)}^{r}}\left(t\right)w^{{\left(i\right)}^{r^{T}}}\left(s\right)\right]=\left[\begin{array}{cccc} \beta_{i} & 0 & 0 & 0\\ 0 & \beta_{i} & 0 & 0\\ 0 & 0 & \beta_{i} & 0\\ 0 & 0 & 0 & \beta_{i} \end{array}\right]\delta_{t,s},$$
  with $\beta_{1}=4$, $\beta_{2}=8$, and $\beta_{3}=25$, and independent of u(t). Note that, if $\alpha_{i}=0$, then the noises u(t) and $v^{\left(i\right)}\left(t\right)$ are uncorrelated. In the opposite case, when $\alpha_{i}$ becomes more different from 0, the correlation between u(t) and $v^{\left(i\right)}\left(t\right)$ is stronger.

Moreover, at every sensor *i*, the Bernoulli random variables $\gamma_{\nu }^{\left(i\right)}\left(t\right)$, $\nu =r,\eta ,\eta^{\prime },\eta^{\prime \prime }$, have the constant probabilities $P\left[\gamma_{\nu }^{\left(i\right)}\left(t\right)=1\right]=p_{\nu }^{\left(i\right)}$, for all t∈T.

In this framework, a comparative study between tessarine and quaternion approaches is carried out to evaluate the performance of the proposed filtering, prediction and smoothing algorithms under $T_{1}$ and $T_{2}$ properness conditions. Specifically, besides the filtering, the 3-step prediction and fixed-point smoother at t=20 problems are considered in our simulations.

### 5.1. Study Case 1: $T_{1}$-Proper Systems

Consider the values a=4 in (25) and b=0.9 and c=0.3 in (26), and the Bernoulli probabilities

- $p_{r}^{\left(1\right)}=p_{\eta }^{\left(1\right)}=p_{\eta^{\prime }}^{\left(1\right)}=p_{\eta^{\prime \prime }}^{\left(1\right)}=p_{1}$;
- $p_{r}^{\left(2\right)}=p_{\eta }^{\left(2\right)}=p_{\eta^{\prime }}^{\left(2\right)}=p_{\eta^{\prime \prime }}^{\left(2\right)}=p_{2}$;
- $p_{r}^{\left(3\right)}=p_{\eta }^{\left(3\right)}=p_{\eta^{\prime }}^{\left(3\right)}=p_{\eta^{\prime \prime }}^{\left(3\right)}=p_{3}$.

Note that, under these conditions, both x(t) and $y^{\left(i\right)}\left(t\right)$, i=1,2,3, are jointly $T_{1}$-proper.

For the purpose of comparison, the error variances of both $T_{1}$ and QSL estimators have been computed for different Bernoulli probabilities $p_{i}$, i=1,2,3. We denote the QSL error variances by $P_{QSL}\left(t|s\right)$. Then, as a performance measure, we compute the difference between the $T_{1}$ and QSL error variances associated to the filter, $DE_{1}\left(t|t\right)=P_{QSL}\left(t|t\right)-P_{1}\left(t|t\right)$, the 3-step predictor, $DE_{1}\left(t+3|t\right)=P_{QSL}\left(t+3|t\right)-P_{1}\left(t+3|t\right)$, and the fixed-point smoother at t=20, $DE_{1}\left(20|t\right)=P_{QSL}\left(20|t\right)-P_{1}\left(20|t\right)$, for t>20.

**Figure 1:**
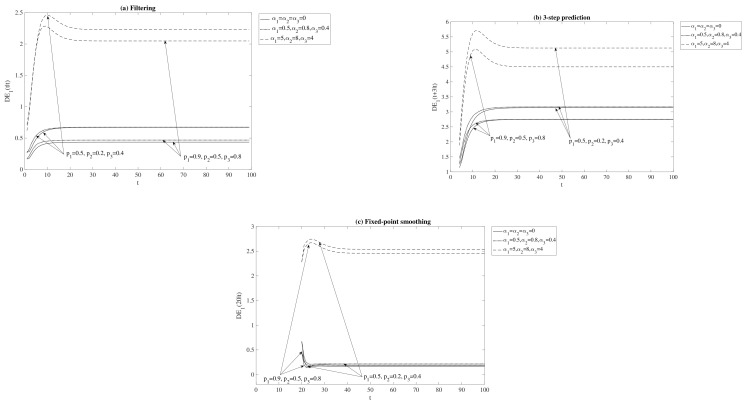
Difference between QSL and $T_{1}$ error variances for the problem of (**a**) filtering, (**b**) 3-step prediction and (**c**) fixed-point smoothing.

Firstly, these differences are displayed in Figure 1 considering different degrees of correlations between the state and measurement noises: independent noises ($\alpha_{1}=\alpha_{2}=\alpha_{3}=0$), low correlations ($\alpha_{1}=0.5$, $\alpha_{2}=0.8$, $\alpha_{3}=0.4$), and high correlations ($\alpha_{1}=5$, $\alpha_{2}=8$, $\alpha_{3}=4$) and two levels of uncertainties: high delay probabilities (case $p_{1}=0.5$, $p_{2}=0.2$, $p_{3}=0.4$) and low delay probabilities (case $p_{1}=0.9$, $p_{2}=0.5$, $p_{3}=0.8$). As we can see, in all situations these differences are positive, which indicate that the proposed $T_{1}$ estimators outperform the QSL estimators. Moreover, this superiority in performance increases when the correlation between the system noises is higher. With respect to the levels of uncertainties, a better behavior of the $T_{1}$ estimators over the QSL counterparts is generally observed in the scenario of high delays probabilities, i.e., when the Bernoulli probabilities are smaller.

**Figure 2:**
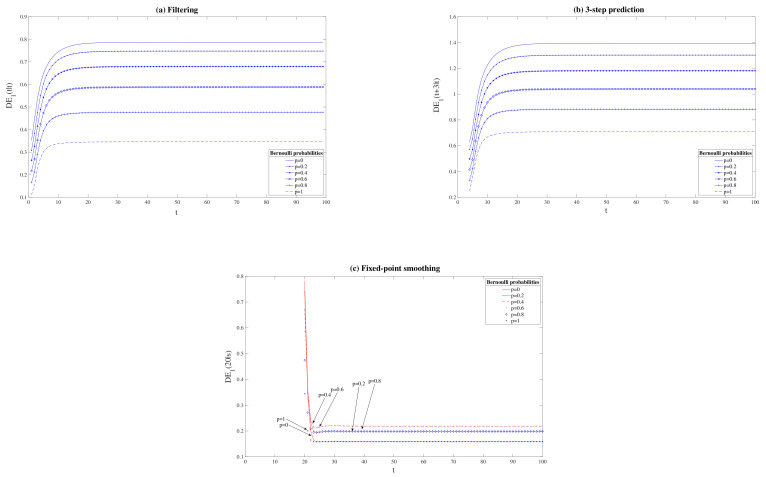
Difference between QSL and $T_{1}$ error variances for the problem of (**a**) filtering, (**b**) 3-step prediction and (**c**) fixed-point smoothing.

Next, in order to evaluate the performance of the proposed estimators versus the probability of delay, we consider the same Bernoulli probabilities in the three sensors $\left(p_{1}=p_{2}=p_{3}=p\right)$, and the difference between the $T_{1}$ and QSL error variances are computed for different values of *p*. Figure 2 illustrates these differences for p=0,0.2,0.4,0.6,0.8,1. In these figures, the superiority in performance of $T_{1}$ estimators over QSL estimators is confirmed since $DE_{1}>0$ in every case. Additionally, in the filtering and prediction problems it is observed that this superiority is higher for the smallest Bernoulli probabilities, i.e., when the delay probabilities are greater. On the other hand, in the fixed-point smoothing problem, a similar behavior for Bernoulli probabilities *p* and 1−p is obtained, the advantages of the $T_{1}$ smoothing algorithm being higher than the QSL one at intermediate values of *p* (case p=0.4 and p=0.6). These results are examined in detail below.

Our aim now is to analyze the benefits of our $T_{1}$ estimation algorithms in terms of the Bernoulli probabilities of the three sensors *p*. In this analysis, different values of *c* in (26) are also considered. Then, the means of the difference between the $T_{1}$ and QSL filtering, prediction, and fixed-point smoothing error variances have been computed as

**Figure 3:**
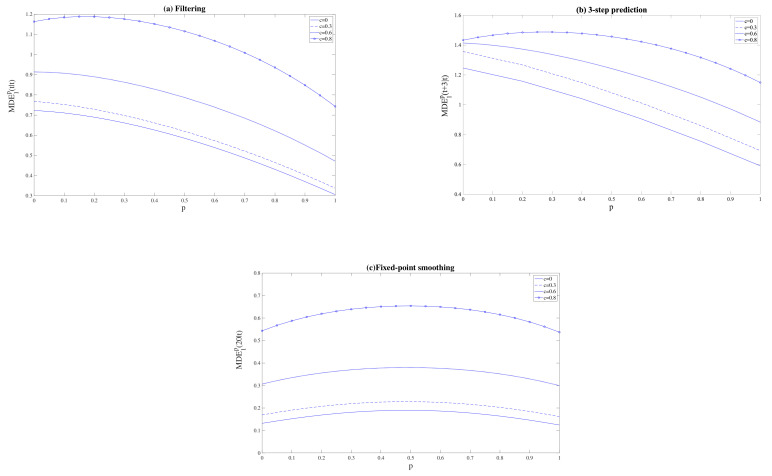
Mean of the difference between QSL and $T_{1}$ error variances for the problem of (**a**) filtering, (**b**) 3-step prediction, and (**c**) fixed-point smoothing.

- Filtering problem: $MDE_{1}^{p}\left(t|t\right)=\frac{1}{100}\sum_{t=1}^{100}DE_{1}^{p}\left(t|t\right)$;
- 3-step prediction problem: $MDE_{1}^{p}\left(t+3|t\right)=\frac{1}{97}\sum_{t=1}^{97}DE_{1}^{p}\left(t+3|t\right)$;
- Fixed-point smoothing problem: $MDE_{1}^{p}\left(20|t\right)=\frac{1}{80}\sum_{t=1}^{80}DE_{1}^{p}\left(20|t\right)$;

for *p* varying from 0 to 1 and the values of c=0,0.3,0.6, and 0.8, and where $DE_{1}^{p}\left(t|t\right)$, $DE_{1}^{p}\left(t+3|t\right)$ and $DE_{1}^{p}\left(20|t\right)$ denote the difference between the $T_{1}$ and QSL filtering, 3-step prediction, and fixed-point smoothing error variances, respectively, for a value of the Bernoulli probability *p*. Note that in the case c=0, the noise u(t) is, besides being $T_{1}$-proper, Q-proper, and a higher value of *c* means that the noise u(t) moves further away from the Q-properness condition. The results of this analysis are depicted in Figure 3 where, on the one hand, we can clearly observe how the best performance of $T_{1}$ filtering and prediction estimators over the QSL counterparts is obtained for the smallest Bernoulli probabilities. Specifically, except for the case c=0.8, the maximum difference between $T_{1}$ and QSL errors is achieved when the Bernoulli probability takes the value 0, i.e., when only one-step delay exists in the measurements. However, in the fixed-point smoothing problem $T_{1}$ is more advantageous when the Bernoulli probabilities *p* tend to 0.5. On the other hand, in every case, the superiority of our $T_{1}$ estimation algorithms is more evident as the parameter *c* in (26) grows, i.e., the noise u(t) is further away from the Q-properness condition.

### 5.2. Study Case 2: $T_{2}$-Proper Systems

Consider the values a=6 in (25), b=c=0.3 in (26), and the Bernoulli probabilities for the three sensors as in Section 5.1. Note that, under these conditions, both x(t) and $y^{\left(i\right)}\left(t\right)$, i=1,2,3, are jointly $T_{2}$-proper.

Thus, we are interested in comparing the behavior of $T_{2}$ centralized fusion estimators with their counterparts in the quaternion domain, i.e., the quaternion semi-widely linear (QSWL) estimators. For this purpose, the $T_{2}$ and QSWL error variances, $P_{2}\left(t|s\right)$ and $P_{QSWL}\left(t|s\right)$, respectively, have been computed by considering different Bernoulli probabilities for the three sensors.

Specifically, we consider the filtering, the 3-step prediction, and the fixed-point smoothing problems at t=20, and, as a measure of comparison, we use the difference between both QSWL and $T_{2}$ error variances, which are defined as $DE_{2}\left(t|t\right)=P_{QSWL}\left(t|t\right)-P_{2}\left(t|t\right)$ (filtering), $DE_{2}\left(t+3|t\right)=P_{QSWL}\left(t+3|t\right)-P_{2}\left(t+3|t\right)$ (3-step prediction), and $DE_{2}\left(20|t\right)=P_{QSWL}\left(20|t\right)-P_{2}\left(20|t\right)$ (fixed-point smoothing).

**Figure 4:**
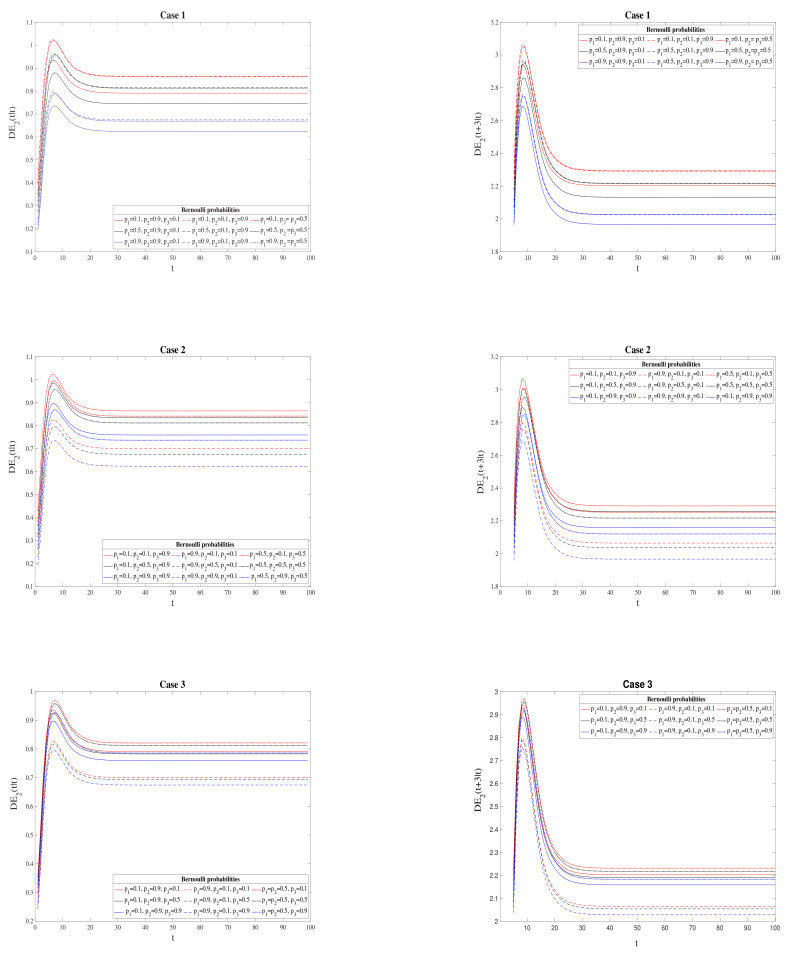
Difference between QSWL and $T_{2}$ error variances for the problem of filtering (left column) and 3-step prediction (right column) for Cases 1–3.

**Figure 5:**
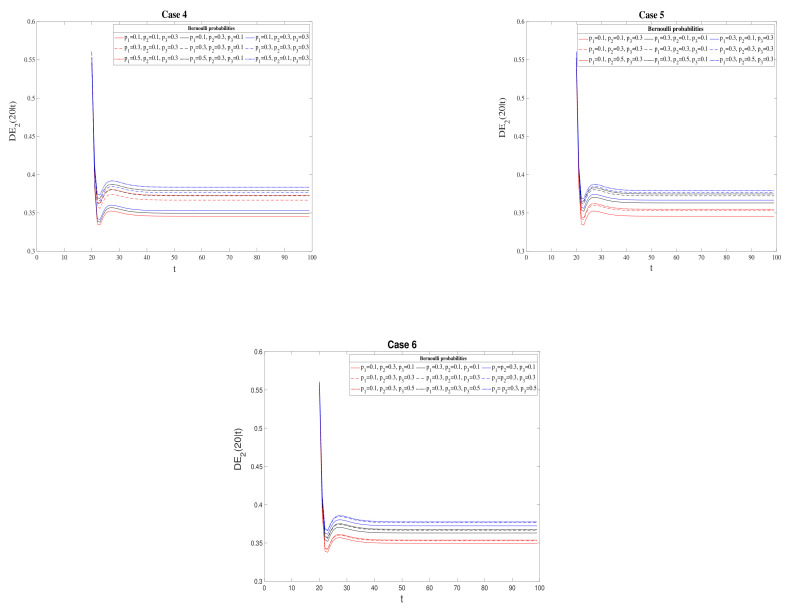
Difference between QSWL and $T_{2}$ error variances for the fixed-point smoothing problem for Cases 4–6.

Figure 4 and Figure 5 compare the difference between QSWL and $T_{2}$ centralized estimation error variances for different Bernoulli probabilities $p_{1}$, $p_{2}$ and $p_{3}$. Specifically, Figure 4 analyzes the filtering and 3-step prediction error variance differences $DE_{2}\left(t|t\right)$ and $DE_{2}\left(t+3|t\right)$ for the following cases:

1. Case 1: for values of $p_{1}=0.1,0.5,0.9$ in three situations: $p_{2}=0.9$ and $p_{3}=0.1$, $p_{2}=0.1$ and $p_{3}=0.9$, and $p_{2}=p_{3}=0.5$;
2. Case 2: for values of $p_{2}=0.1,0.5,0.9$ in three situations: $p_{1}=0.9$ and $p_{3}=0.1$, $p_{1}=0.1$ and $p_{3}=0.9$, and $p_{1}=p_{3}=0.5$;
3. Case 3: for values of $p_{3}=0.1,0.5,0.9$ in three situations: $p_{1}=0.9$ and $p_{2}=0.1$, $p_{1}=0.1$ and $p_{2}=0.9$, and $p_{1}=p_{2}=0.5$.

It should be highlighted that similar results are obtained with any other combination of Bernoulli probabilities $p_{i}$, i=1,2,3.

From these figures, we can reaffirm that $T_{2}$ processing is a better approach than the QSWL processing in terms of performance ($DE_{2}>0$). Moreover, in the filtering and 3-step prediction problems (Figure 4), this fact is more evident when the probabilities of the Bernoulli variables decrease (that is, the delay probabilities increase).

The differences between both QSWL and $T_{2}$ error variances for the fixed-point smoothing problem are illustrated in Figure 5. Note that, since the behavior of the differences between QSWL and $T_{2}$ fixed-point smoothing errors is similar for Bernoulli probabilities values $p_{i}$ and $1-p_{i}$, these differences are analyzed in the following cases:

1. Case 4: for values of $p_{1}=0.1,0.3,0.5$ in three situations: $p_{2}=0.1$ and $p_{3}=0.3$, $p_{2}=0.3$ and $p_{3}=0.1$, and $p_{2}=p_{3}=0.3$.
2. Case 5: for values of $p_{2}=0.1,0.3,0.5$ in three situations: $p_{1}=0.1$ and $p_{3}=0.3$, $p_{1}=0.3$ and $p_{3}=0.1$, and $p_{1}=p_{3}=0.3$.
3. Case 6: for values of $p_{3}=0.1,0.3,0.5$ in three situations: $p_{1}=0.1$ and $p_{2}=0.3$, $p_{1}=0.3$ and $p_{2}=0.1$, and $p_{1}=p_{2}=0.3$.

In every situation, the better behavior of $T_{2}$ processing over the QSWL processing is verified, and also this superiority increases when the Bernoulli probabilities tends to 0.5, i.e., when there is a similar chance of receiving updated and delayed information.

## 6. Discussion

From among the different sensor fusion methods, it is the centralized fusion techniques that provide the optimal estimators from measurements of all sensors. Nevertheless, to avoid the computational load involved in these estimates, especially in systems with a large number of sensors, suboptimum estimation algorithms have been traditionally designed by using a decentralized fusion approach. This paper has overcome the above computational difficulties without renouncing to obtain the optimal solution, by considering hypercomplex algebras. Quaternions and, more recently, tessarines are the most usual 4D hypercomplex algebra employed in signal processing. Commonly, since both quaternions and tessarines are isomorfic spaces to $R^{4}$, they involve the same computational complexity. Interestingly, under properness conditions, this complexity in terms of dimension is reduced to a half for QSWL and $T_{2}$-proper methods and four times for QSL and $T_{1}$-proper methods, which leads to a significant reduction in the computational load of our algorithms. Precisely, it is in this context that the use of hypercomplex algebras becomes an ideal tool with computational advantages over the existing methods to address the centralized fusion estimation problem.

In general, neither of these algebras always performs better than the other, and the choice of the most suitable one is conditioned by the characteristics of the signal. Due to the commutativity and reduced computational complexity, the tessarine algebra makes it particularly interesting for our purposes. Thus, under conditions of $T_{k}$-properness, filtering, prediction, and fixed-point smoothing algorithms of reduced dimension have been devised for the estimation of a vectorial tessarine signal based on one-step randomly delayed observations coming from multiple sensors stochastic systems with different delay rates and correlated noises. The reduction of the dimension of the problem under $T_{k}$-properness scenarios makes it possible for these algorithms to facilitate the computation of the optimal estimates with a lower computational cost in comparison with the real processing approach. It should be highlighted that this computational saving cannot be attained in the real field.

The good performance of the algorithms proposed has been experimentally illustrated by means of two simulation examples, where the better behavior of the proposed $T_{k}$ estimates over their counterparts in the quaternion domain under $T_{k}$-properness conditions has been evidenced.

In future research, we will set out to explore the design of decentralized fusion estimation algorithms for hypercomplex signals and investigate the use of new hypercomplex algebras in this field.

## Figures and Tables

**Table:** 

	
	
	

## Data Availability

Not applicable.

## Appendix A. Proof of Theorem 1

The proof is based on the innovation technique. Consider the one-state delay model:

(A1) $$\begin{array}{cc} \; & \bar{x}\left(t+1\right)=\bar{\Phi }\left(t\right)\bar{x}\left(t\right)+\bar{u}\left(t\right),\;\;t\geq 0\\ \; & y_{k}\left(t\right)={\tilde{D}}_{k}^{\gamma }\left(t\right)\bar{C}\bar{x}\left(t\right)+{\tilde{D}}_{k}^{\left(1-\gamma \right)}\left(t\right)\bar{C}\bar{x}\left(t-1\right)+{\tilde{D}}_{k}^{\gamma }\left(t\right)\vec{v}\left(t\right)+{\tilde{D}}_{k}^{\left(1-\gamma \right)}\left(t\right)]\vec{v}\left(t-1\right),\;t\geq 1 \end{array}$$

and define the innovations as $\varepsilon_{k}\left(t\right)=y_{k}\left(t\right)-{\hat{y}}_{k}\left(t|t-1\right)$.

In order to simplify the proof of Theorem 1, the following results have been previously established.

### Appendix A.1. Preliminary Results

The following property, stated without proof, about the correlations between the innovations $\varepsilon_{k}\left(t\right)$ and the augmented state $\bar{x}\left(t\right)$ and the noises $\bar{u}\left(t\right)$ and $v_{k}\left(t\right)$, will be useful in the proof of Theorem 1.

**Property** **A1.**
*Given the system (A1), and under the Assumptions 1-4, the following correlations hold:*

(1) *$E\left[\bar{u}\left(t\right)\varepsilon_{k}^{H}\left(t\right)\right]=\bar{S}\left(t\right){\tilde{\Pi }}_{k}^{\gamma^{H}}\left(t\right)$.*
(2) *$E\left[\bar{u}\left(t\right)\varepsilon_{k}^{H}\left(s\right)\right]=0_{4n\times m}$, for t>s.*
(3) *$E\left[\bar{v}\left(t\right)\varepsilon_{k}^{H}\left(t\right)\right]=\bar{R}\left(t\right){\tilde{\Pi }}_{k}^{\gamma^{H}}\left(t\right)$.*
(4) *$E\left[\bar{v}\left(t\right)\varepsilon_{k}^{H}\left(s\right)\right]=0_{4Rn\times m}$, for t>s.*

Moreover, the following results will be of interest in the derivation of the formulas given in Theorem 1.

**Lemma** **A1.**
*Denote $\Delta {\tilde{D}}_{k}^{\gamma }\left(t\right)={\tilde{D}}_{k}^{\gamma }\left(t\right)-{\tilde{\Pi }}_{k}^{\vec{\gamma }}\left(t\right)$ and $\Delta {\tilde{D}}_{k}^{\left(1-\gamma \right)}\left(t\right)={\tilde{D}}_{k}^{\left(1-\gamma \right)}\left(t\right)-{\tilde{\Pi }}_{k}^{\left(1-\gamma \right)}\left(t\right)$. For any tessarine random vectors $\alpha_{1}\left(t\right),\alpha_{2}\left(t\right)\in T^{4Rn}$ and $\beta \left(t\right)\in T^{q}$, for any dimension q, the following relations hold:*

1 *$E\left[\Delta {\tilde{D}}_{k}^{\gamma }\left(t\right)\alpha_{1}\left(t\right)\alpha_{2}^{H}\left(s\right)\Delta {\tilde{D}}_{k}^{\gamma^{H}}\left(t\right)\right]=L_{k}\left\{\mathrm{Cov}\left({\vec{\gamma }}^{r}\left(t\right)\right)\circ \left({\bar{L}}^{H}E\left[\alpha_{1}\left(t\right){\bar{\alpha_{2}}}^{H}\left(s\right)\right]\bar{L}\right)\right\}L_{k}^{H}$.*
2 *$E\left[\Delta {\tilde{D}}_{k}^{\gamma }\left(t\right)\alpha_{1}\left(t\right)\alpha_{2}^{H}\left(s\right){\tilde{D}}_{k}^{\gamma^{H}}\left(t\right)\right]=L_{k}\left\{\mathrm{Cov}\left({\vec{\gamma }}^{r}\left(t\right)\right)\circ \left({\bar{L}}^{H}E\left[\alpha_{1}\left(t\right)\alpha_{2}^{H}\left(s\right)\right]\bar{L}\right)\right\}L_{k}^{H}$.*
3 *$E\left[\Delta {\tilde{D}}_{k}^{\gamma }\left(t\right)\alpha_{1}\left(t\right)\alpha_{2}^{H}\left(s\right){\tilde{D}}_{k}^{{\left(1-\gamma \right)}^{H}}\left(t\right)\right]=-L_{k}\left\{\mathrm{Cov}\left({\vec{\gamma }}^{r}\left(t\right)\right)\circ \left({\bar{L}}^{H}E\left[\alpha_{1}\left(t\right)\alpha_{2}^{H}\left(s\right)\right]\bar{L}\right)\right\}L_{k}^{H}$.*
4 *$E\left[\Delta {\tilde{D}}_{k}^{\gamma }\left(t\right)\alpha_{1}\left(t\right)\beta^{H}\left(s\right)\right]=0_{m\times q}$.*
5 *$E\left[{\tilde{D}}_{k}^{\gamma }\left(t\right)\alpha_{1}\left(t\right)\alpha_{2}^{H}\left(s\right){\tilde{D}}_{k}^{\gamma^{H}}\left(t\right)\right]=L_{k}\left\{E\left[{\vec{\gamma }}^{r}\left(t\right){\vec{\gamma }}^{r^{T}}\left(t\right)\right]\circ \left({\bar{L}}^{H}E\left[\alpha_{1}\left(t\right)\alpha_{2}^{H}\left(s\right)\right]\bar{L}\right)\right\}L_{k}^{H}$.*
6 *$E\left[{\tilde{D}}_{k}^{\gamma }\left(t\right)\alpha_{1}\left(t\right)\alpha_{2}^{H}\left(s\right){\tilde{D}}_{k}^{{\left(1-\gamma \right)}^{H}}\left(t\right)\right]=\;L_{k}\left\{E\left[{\vec{\gamma }}^{r}\left(t\right){\left(1_{4Rn}-{\vec{\gamma }}^{r}\left(t\right)\right)}^{T}\right]\circ \left({\bar{L}}^{H}E\left[\alpha_{1}\left(t\right)\alpha_{2}^{H}\left(s\right)\right]\bar{L}\right)\right\}L_{k}^{H}$.*
7 *$E\left[{\tilde{D}}_{k}^{\left(1-\gamma \right)}\left(t\right)\alpha_{1}\left(t\right)\alpha_{2}^{H}\left(s\right){\tilde{D}}_{k}^{{\left(1-\gamma \right)}^{H}}\left(t\right)\right]=\;L_{k}\left\{E\left[\left(I_{m}-{\vec{\gamma }}^{r}\left(t\right)\right){\left(I_{m}-{\vec{\gamma }}^{r}\left(t\right)\right)}^{T}\right]\circ \left({\bar{L}}^{H}E\left[\alpha_{1}\left(t\right)\alpha_{2}^{H}\left(s\right)\right]\bar{L}\right)\right\}L_{k}^{H}$.*

**Proof.** The proof is immediate from (1) and taking into account that ${\tilde{D}}_{k}^{\gamma }\left(t\right)=L_{k}\mathrm{diag}\left({\vec{\gamma }}^{r}\left(t\right)\right){\bar{L}}^{H}$ and ${\tilde{D}}_{k}^{\left(1-\gamma \right)}\left(t\right)=L_{k}\mathrm{diag}\left(1_{4Rn}-{\vec{\gamma }}^{r}\left(t\right)\right){\bar{L}}^{H}$. □

### Appendic A.2. Expressions in Theorem 1

Although tessarine algebra is not a Hilbert space, the existence and uniqueness of the projection of an element on the set of measurements $\left\{y_{k}\left(1\right),\ldots ,y_{k}\left(s\right)\right\}$, for k=1,2, is guaranteed ([23]). Now, from Theorem 3 of [23], we obtain

(A2) $$\hat{\bar{x}}\left(t|t\right)=\hat{\bar{x}}\left(t|t-1\right)+{\tilde{L}}_{k}\left(t\right)\varepsilon_{k}\left(t\right),$$

with ${\tilde{L}}_{k}\left(t\right)={\tilde{\Theta }}_{k}\left(t\right)\Omega_{k}^{-1}\left(t\right)$, where ${\tilde{\Theta }}_{k}\left(t\right)=E\left[\bar{x}\left(t\right)\varepsilon_{k}^{H}\left(t\right)\right]$ and $\Omega_{k}\left(t\right)=E\left[\varepsilon_{k}\left(t\right)\varepsilon_{k}^{H}\left(t\right)\right]$. Then, by applying $T_{k}$-properness conditions, (10) is directly devised.

Taking projections on both sides of the state and observation equations in (A1) onto the linear space spanned by $\left\{\varepsilon_{k}\left(1\right),\ldots ,\varepsilon_{k}\left(t-1\right)\right\}$, and using Property A1, we have

(A3) $$\hat{\bar{x}}\left(t+1|t\right)=\bar{\Phi }\left(t\right)\hat{\bar{x}}\left(t|t\right)+{\tilde{H}}_{k}\left(t\right)\varepsilon_{k}\left(t\right)$$

(A4) $${\hat{y}}_{k}\left(t|t-1\right)={\tilde{\Pi }}_{k}^{\vec{\gamma }}\left(t\right)\bar{C}\hat{\bar{x}}\left(t|t-1\right)+{\tilde{\Pi }}_{k}^{\left(1-\gamma \right)}\left(t\right)\left[\bar{C}\hat{\bar{x}}\left(t-1|t-1\right)+\hat{\bar{v}}\left(t-1|t-1\right)\right]$$

where ${\tilde{H}}_{k}\left(t\right)=\bar{S}\left(t\right){\tilde{\Pi }}_{k}^{\gamma^{H}}\left(t\right)\Omega_{k}^{-1}\left(t\right)$ and $\hat{\bar{v}}\left(t|t\right)={\tilde{G}}_{k}\left(t\right)\varepsilon_{k}\left(t\right)$, with ${\tilde{G}}_{k}\left(t\right)=\bar{R}\left(t\right){\tilde{\Pi }}_{k}^{\gamma^{H}}\left(t\right)\Omega_{k}^{-1}\left(t\right)$.

Then, (11) follows from (A3) and the $T_{k}$-properness conditions on $\bar{\Phi }\left(t\right)$ and ${\tilde{\Pi }}_{k}^{\vec{\gamma }}\left(t\right)$ established in Proposition 1 and Remark 3. Likewise, (12) is easily obtained from (A4).

Consider now the gain matrix ${\tilde{L}}_{k}\left(t\right)={\tilde{\Theta }}_{k}\left(t\right)\Omega_{k}^{-1}\left(t\right)$ in (A2). Denote the prediction error and its covariance matrix as $\bar{\epsilon }\left(t|t-1\right)=\bar{x}\left(t\right)-\hat{\bar{x}}\left(t|t-1\right)$ and $\bar{P}\left(t|t-1\right)=E\left[\bar{\epsilon }\left(t|t-1\right){\bar{\epsilon }}^{H}\left(t|t-1\right)\right]$, respectively. Then, by applying (A1) and (A4), $\bar{\epsilon }\left(t|t-1\right)⊥\hat{\bar{x}}\left(t|t-1\right)$, Property 3 and Property A1, we have

(A5) $$\begin{array}{c} {\tilde{\Theta }}_{k}\left(t\right)=\bar{P}\left(t|t-1\right){\bar{C}}^{T}{\tilde{\Pi }}_{k}^{\gamma^{H}}\left(t\right)+\bar{\Phi }\left(t-1\right)\bar{P}\left(t-1|t-1\right){\bar{C}}^{T}{\tilde{\Pi }}_{k}^{{\left(1-\gamma \right)}^{H}}\left(t\right)+\bar{S}\left(t-1\right){\tilde{\Pi }}_{k}^{{\left(1-\gamma \right)}^{H}}\left(t\right)\\ -{\tilde{H}}_{k}\left(t-1\right){\tilde{\Theta }}_{k}^{H}\left(t-1\right){\bar{C}}^{T}{\tilde{\Pi }}_{k}^{{\left(1-\gamma \right)}^{H}}\left(t\right)-\bar{\Phi }\left(t-1\right){\tilde{\Theta }}_{k}\left(t-1\right){\tilde{G}}_{k}^{H}\left(t-1\right){\tilde{\Pi }}_{k}^{{\left(1-\gamma \right)}^{H}}\left(t\right)\\ -{\tilde{H}}_{k}\left(t-1\right)\Omega_{k}\left(t-1\right){\tilde{G}}_{k}^{H}\left(t-1\right){\tilde{\Pi }}_{k}^{{\left(1-\gamma \right)}^{H}}\left(t\right),\;t>1 \end{array}$$

and thus, the recursive expression (13) is directly obtained from (A5), by applying the $T_{k}$-properness conditions on $\bar{\Phi }\left(t\right)$, and denoting by $P_{k}\left(t|t-1\right)$ the first m×m submatrix of $\bar{P}\left(t|t-1\right)$.

Next, we devise the expression for the innovation covariance matrix (14). For this purpose, the innovations are rewritten in the following form

(A6) $$\begin{array}{c} \varepsilon_{k}\left(t\right)=\Delta {\tilde{D}}_{k}^{\gamma }\left(t\right)\bar{C}\bar{x}\left(t\right)+{\tilde{\Pi }}_{k}^{\vec{\gamma }}\left(t\right)\bar{C}\bar{\epsilon }\left(t|t-1\right)-\Delta {\tilde{D}}_{k}^{\gamma }\left(t\right)\bar{C}\bar{x}\left(t-1\right)+{\tilde{\Pi }}_{k}^{\left(1-\gamma \right)}\left(t\right)\bar{C}\bar{\epsilon }\left(t-1|t-1\right)\\ +{\tilde{D}}_{k}^{\gamma }\left(t\right)\vec{v}\left(t\right)+{\tilde{D}}_{k}^{\left(1-\gamma \right)}\left(t\right)\vec{v}\left(t-1\right)-{\tilde{\Pi }}_{k}^{\left(1-\gamma \right)}\left(t\right){\tilde{G}}_{k}\left(t-1\right)\varepsilon_{k}\left(t-1\right). \end{array}$$

From (A3), the prediction error $\bar{\epsilon }\left(t+1|t\right)$ can be expressed as

(A7) $$\bar{\epsilon }\left(t+1|t\right)=\bar{\Phi }\left(t\right)\bar{\epsilon }\left(t|t\right)+\bar{u}\left(t\right)-{\tilde{H}}_{k}\left(t\right)\varepsilon_{k}\left(t\right).$$

As a consequence, from (A7), using Property 3 and Property A1, and taking into account that $\bar{\epsilon }\left(t|t\right)⊥\varepsilon_{k}\left(t\right)$, we have

(A8) $$\begin{array}{ccc} & & E\left[\bar{u}\left(t\right){\bar{\epsilon }}^{H}\left(t|t\right)\right]=-{\tilde{H}}_{k}\left(t\right){\tilde{\Theta }}_{k}^{H}\left(t\right), \end{array}$$

(A9) $$\begin{array}{ccc} & & E\left[\bar{\epsilon }\left(t+1|t\right){\bar{\epsilon }}^{H}\left(t|t\right)\right]=\bar{\Phi }\left(t\right)\bar{P}\left(t|t\right)-{\tilde{H}}_{k}\left(t\right){\tilde{\Theta }}_{k}^{H}\left(t\right), \end{array}$$

(A10) $$\begin{array}{ccc} & & E\left[\bar{\epsilon }\left(t|t\right){\bar{v}}^{H}\left(t\right)\right]=-{\tilde{\Theta }}_{k}\left(t\right){\tilde{G}}_{k}^{H}\left(t\right), \end{array}$$

(A11) $$\begin{array}{ccc} & & E\left[\bar{\epsilon }\left(t|t\right){\bar{v}}^{H}\left(t+1\right)\right]=0_{4n\times 4Rn}, \end{array}$$

(A12) $$\begin{array}{ccc} & & E\left[\bar{\epsilon }\left(t+1|t\right){\bar{v}}^{H}\left(t\right)\right]=\bar{S}\left(t\right)-\bar{\Phi }\left(t\right){\tilde{\Theta }}_{k}\left(t\right){\tilde{G}}_{k}^{H}\left(t\right)-{\tilde{H}}_{k}\left(t\right){\tilde{\Omega }}_{k}\left(t\right){\tilde{G}}_{k}^{H}\left(t\right), \end{array}$$

(A13) $$\begin{array}{ccc} & & E\left[\bar{\epsilon }\left(t+1|t\right){\bar{v}}^{H}\left(t+1\right)\right]=0_{4n\times 4Rn}. \end{array}$$

Then, the expression (14) for the innovation covariance matrix is obtained from (A6), by using Lemma A1, Property 3, Property A1, (A9)–(A13), $\bar{\epsilon }\left(t+1|t\right)⊥\varepsilon_{k}\left(t\right)$, and by applying $T_{k}$ properness conditions. Furthermore, the recursion of $\bar{D}\left(t\right)=E\left[\bar{x}\left(t\right){\bar{x}}^{H}\left(t\right)\right]$ given in (16) is a direct consequence of the augmented state equation in system (A1). In a similar way, Equation (15) follows.

In the following step, consider the filtering error covariance matrix $\bar{P}\left(t|t\right)=E\left[\bar{\epsilon }\left(t|t\right){\bar{\epsilon }}^{H}\left(t|t\right)\right]$ with $\bar{\epsilon }\left(t|t\right)=\bar{x}\left(t\right)-\hat{\bar{x}}\left(t|t\right)$. From (A2), we directly obtain that $\bar{P}\left(t|t\right)=\bar{P}\left(t|t-1\right)-{\tilde{\Theta }}_{k}\left(t\right)\Omega_{k}^{-1}\left(t\right){\tilde{\Theta }}_{k}^{H}\left(t\right)$ and thus (17) holds by virtue of $T_{k}$ properness conditions.

Finally, from (A7), and taking into consideration that $\bar{\epsilon }\left(t|t\right)⊥\varepsilon_{k}\left(t\right)$, (A8), and Property A1, we have

$$\begin{array}{c} \bar{P}\left(t+1|t\right)=\bar{\Phi }\left(t\right)\bar{P}\left(t|t\right){\bar{\Phi }}^{H}\left(t\right)-{\tilde{H}}_{k}\left(t\right){\tilde{\Theta }}_{k}^{H}\left(t\right){\bar{\Phi }}^{H}\left(t\right)\\ -\bar{\Phi }\left(t\right){\tilde{\Theta }}_{k}\left(t\right){\tilde{H}}_{k}^{H}\left(t\right)-{\tilde{H}}_{k}\left(t\right)\Omega_{k}\left(t\right){\tilde{H}}_{k}^{H}\left(t\right)+\bar{Q}\left(t\right). \end{array}$$

From $T_{k}$ properness conditions (18) follows.

## Appendix A. Proof of Theorem 2

From the projection of x(t+τ) onto the linear space spanned by $\left\{\varepsilon_{k}\left(1\right),\cdots ,\varepsilon_{k}\left(t\right)\right\}$, we have

$$\hat{\bar{x}}\left(t+\tau |t\right)=\Phi \left(t+\tau -1\right)\hat{\bar{x}}\left(t+\tau -1|t\right),\tau \geq 2$$

Then, from $T_{k}$ properness conditions, (19) holds.

Finally, from (19), it is clear that the prediction error covariance matrix $P_{k}\left(t+\tau |t\right)$ satisfies the recursive expression (20).

## Appendix B. Proof of Theorem 3

By projecting the state x(t) onto the linear space spanned by $\left\{\varepsilon_{k}\left(1\right),\cdots ,\varepsilon_{k}\left(s\right)\right\}$, we have

(A14) $$\hat{\bar{x}}\left(t|s\right)=\hat{\bar{x}}\left(t|s-1\right)+{\tilde{L}}_{k}\left(t,s\right)\epsilon_{k}\left(s\right),\;\;t<s$$

with ${\tilde{L}}_{k}\left(t,s\right)={\tilde{\theta }}_{k}\left(t,s\right)\Omega_{k}^{-1}\left(s\right)$, where ${\tilde{\theta }}_{k}\left(t,s\right)=E\left[\bar{x}\left(t\right)\epsilon_{k}^{H}\left(s\right)\right]$. Then, (21) is directly derived from (A8), by applying $T_{k}$ properness conditions.

Consider the matrix ${\tilde{\theta }}_{k}\left(t,s\right)$. From (12) and (8), we have

$$\begin{array}{c} {\tilde{\theta }}_{k}\left(t,s\right)=E\left[\bar{x}\left(t\right){\bar{\epsilon }}^{H}\left(s|s-1\right)\right]{\bar{C}}^{T}{\tilde{\Pi }}_{k}^{\vec{\gamma }}\left(s\right)+E\left[\bar{x}\left(t\right){\bar{\epsilon }}^{H}\left(s-1|s-1\right)\right]{\bar{C}}^{T}\left(I_{m}-{\tilde{\Pi }}_{k}^{\vec{\gamma }}\left(s\right)\right)\\ -{\tilde{\theta }}_{k}\left(t,s-1\right){\tilde{G}}_{k}^{H}\left(s-1\right)\left(I_{m}-{\tilde{\Pi }}_{k}^{\vec{\gamma }}\left(s\right)\right) \end{array}$$

Let us define the matrix $\bar{E}\left(t,s\right)=E\left[\bar{x}\left(t\right){\bar{\epsilon }}^{H}\left(s|s\right)\right]$. Thus, from (A1) and (A3), it follows that

(A15) $$\begin{array}{c} {\tilde{\theta }}_{k}\left(t,s\right)=\left[\bar{E}\left(t,s-1\right){\bar{\Phi }}^{H}\left(s-1\right)-{\tilde{\theta }}_{k}\left(t,s-1\right){\tilde{H}}_{k}^{H}\left(s-1\right)\right]{\bar{C}}^{T}{\tilde{\Pi }}_{k}^{\vec{\gamma }}\left(s\right)\\ +\left[\bar{E}\left(t,s-1\right){\bar{C}}^{T}-{\tilde{\theta }}_{k}\left(t,s-1\right){\tilde{G}}_{k}^{H}\left(s-1\right)\right]\left(I_{m}-{\tilde{\Pi }}_{k}^{\vec{\gamma }}\left(s\right)\right) \end{array}$$

Then, (22) follows from T proper conditions.

In a similar way, from (A1)–(A3) and (A9), $\bar{E}\left(t,s\right)$ is of the form

(A16) $$\begin{array}{c} \bar{E}\left(t,s\right)=\left[\bar{E}\left(t,s-1\right){\bar{\Phi }}^{H}\left(s-1\right)-{\tilde{\theta }}_{k}\left(t,s-1\right)H^{H}\left(s-1\right)\right]\left(I_{m}-{\bar{C}}^{T}{\tilde{\Pi }}_{k}^{\vec{\gamma }}\left(s\right)L^{H}\left(s\right)\right)\\ -\left[\bar{E}\left(t,s-1\right){\bar{C}}^{T}-{\tilde{\theta }}_{k}\left(t,s-1\right)G^{H}\left(s-1\right)\right]\left(I_{m}-{\tilde{\Pi }}_{k}^{\vec{\gamma }}\left(s\right)\right)L^{H}\left(s\right) \end{array}$$

where $\bar{E}\left(t,t\right)=\bar{P}\left(t|t\right)$. Then, (23) follows from $T_{k}$ proper conditions.

Finally, (24) can be easily derived from (21).
